# Timing of Interleukin-4 Stimulation of Macrophages Determines Their Anti-Microbial Activity during Infection with *Salmonella enterica* Serovar Typhimurium

**DOI:** 10.3390/cells12081164

**Published:** 2023-04-14

**Authors:** Natascha Brigo, Emely Neumaier, Christa Pfeifhofer-Obermair, Philipp Grubwieser, Sabine Engl, Sylvia Berger, Markus Seifert, Vera Reinstadler, Herbert Oberacher, Günter Weiss

**Affiliations:** 1 Department of Internal Medicine II, Medical University of Innsbruck, Anichstrasse 35, 6020 Innsbruck, Austria; 2 Christian Doppler Laboratory for Iron Metabolism and Anemia Research, Medical University of Innsbruck, Anichstrasse 35, 6020 Innsbruck, Austria; 3 Institute of Legal Medicine and Core Facility Metabolomics, Medical University of Innsbruck, Muellerstrasse 44, 6020 Innsbruck, Austria

**Keywords:** interleukin-4, arginase 1, interferon-gamma, inducible nitric oxide synthase, *Salmonella enterica* serovar Typhimurium, macrophages, polyamines, ornithine, intracellular bacteria

## Abstract

Priming of macrophages with interferon-gamma (IFNγ) or interleukin-4 (IL-4) leads to polarisation into pro-inflammatory or anti-inflammatory subtypes, which produce key enzymes such as inducible nitric oxide synthase (iNOS) and arginase 1 (ARG1), respectively, and in this way determine host responses to infection. Importantly, L-arginine is the substrate for both enzymes. ARG1 upregulation is associated with increased pathogen load in different infection models. However, while differentiation of macrophages with IL-4 impairs host resistance to the intracellular bacterium *Salmonella enterica* serovar Typhimurium (*S*.tm), little is known on the effects of IL-4 on unpolarised macrophages during infection. Therefore, bone-marrow-derived macrophages (BMDM) from C57BL/6N, Tie2Cre^+/−^ARG1^fl/fl^ (KO), Tie2Cre^−/−^ARG1^fl/fl^ (WT) mice were infected with *S*.tm in the undifferentiated state and then stimulated with IL-4 or IFNγ. In addition, BMDM of C57BL/6N mice were first polarised upon stimulation with IL-4 or IFNγ and then infected with *S*.tm. Interestingly, in contrast to polarisation of BMDM with IL-4 prior to infection, treatment of non-polarised *S*.tm-infected BMDM with IL-4 resulted in improved infection control whereas stimulation with IFNγ led to an increase in intracellular bacterial numbers compared to unstimulated controls. This effect of IL-4 was paralleled by decreased ARG1 levels and increased iNOS expression. Furthermore, the L-arginine pathway metabolites ornithine and polyamines were enriched in unpolarised cells infected with *S*.tm and stimulated with IL-4. Depletion of L-arginine reversed the protective effect of IL-4 toward infection control. Our data show that stimulation of *S*.tm-infected macrophages with IL-4 reduced bacterial multiplication via metabolic re-programming of L-arginine-dependent pathways.

## 1. Introduction

Macrophages are a central component of the innate immune system. They provide pivotal functions in many biological processes, including pathogen containment and anti-microbial effector functions, iron re-circulation, clearance of cellular debris, and tissue repair and remodelling [1,2]. Because of these multifaceted phenotypes, macrophages are highly pleiotropic cells. Their function is determined by their origin but also dependent on their environment: In response to diverse stimuli, which include endogenous signals produced by injury or infection, antigen-specific signals or self-stimulation, they are activated to alter their cellular metabolism, physiology, and thus their specific function. Interestingly, these environmentally induced characteristics can rapidly change during infection or injury. Due to this dynamic nature, they can support an inflammatory process and also support its resolution afterwards. Broadly, macrophages are divided into two functional types: (I) classically activated macrophages (M1), which eliminate invading pathogens via the induction of several antimicrobial effectors, including inducible nitric oxide synthase (iNOS) expression, and (II) alternatively activated macrophages (M2), which support wound healing, inhibit inflammation, and are characterised by high expression of arginase 1 (ARG1) [3,4,5,6,7,8]. Interleukin-4 (IL-4) is the most critical cytokine for the polarisation of macrophages to the anti-inflammatory subtype (M2).

The binding of IL-4 to its receptors leads to the activation of anti-apoptotic and mitogenic signalling cascades, resulting in stimulation of cellular growth and expression of IL-4 target genes [9]. Importantly, IL-4 induces the expression of the M2 key-enzyme ARG1 [10]. ARG1 activity is responsible for the conversion of L-arginine to ornithine, which is further metabolised to polyamines such as putrescine, spermine, and spermidine; all of these are pivotal for extracellular matrix production [11].

In contrast, polarisation of macrophages to the pro-inflammatory M1 phenotype is triggered by the cytokine IFNγ, which is produced by T-cells, NK cells, and NKT-cells [12,13,14]. IFNγ induces several antimicrobial immune functions such as increased antigen presentation, production of inflammatory cytokines or induction of enzymes such as phagocyte oxidase and iNOS, which generate anti-microbial reactive oxygen species (ROS) or reactive nitrogen species (RNS), respectively. ROS and RNS exert direct bactericidal effects and are critical for host control of infection with intracellular pathogens [7,15,16,17].

In case of infection, specific pathogens can prompt macrophages to differentiate either toward a more pro- or anti-inflammatory polarisation state [18,19].

Several studies have shown that changes in iNOS and ARG1 expression are detrimental to the outcome of specific infections. Upregulation of ARG1 is especially associated with an increased pathogen load of *Leishmania* parasites, *Trypanosoma brucei*, *Streptococcus pneumoniae*, *Mycobacterium bovis*, *Mycobacterium tuberculosis*, or *Toxoplasma gondii* [20,21,22,23,24,25,26].

However, little is known on the physiological stage of macrophages during *Salmonella* infections.

*Salmonella* Typhi, a facultative, intracellular Gram-negative bacterium that can lead to a life-threatening disease in humans. Yearly, 200,000 fatalities are caused by infection with this bacterium. *Salmonella enterica* serovar Typhimurium (*S*.tm), the mouse counterpart to *Salmonella* Typhi, induces self-limiting gastroenteritis in humans but systemic infection in mice [27,28]. Like other intracellular pathogens, *S*.tm can manipulate phagosome maturation by inhibiting the fusion of lysosomes and phagosomes. This allows the bacterium to evade antimicrobial function by macrophages [29,30].

In a mouse model of chronic *S*.tm infection, the initial immune response to infection has been characterized as a pro-inflammatory one, which over time is succeeded by anti-inflammatory host responses. Specifically, anti-inflammatory M2 macrophages become the main reservoir for *Salmonella* in the later stages of infection [31,32,33]. Interestingly, control of *Salmonella*-induced septicaemia in mice is not improved when ARG1 is genetically deleted or pharmacologically blocked [15].

In this current study the influence of IL-4- and IFNγ-dependent polarisation (pre-stimulation) or stimulation of non-polarised (post-stimulation) macrophages upon infection with *Salmonella enterica* serovar Typhimurium was analysed. In most studies published so far, pre-stimulation conditions were used to analyse the role of macrophage polarisation in the course of bacterial infection. However, under physiological conditions non-polarized macrophages are challenged with bacteria and subsequently exposed to cytokines released during the early phase of infection.

Applying such a condition, we could show that un-polarised macrophages which are exposed to cytokines after an infection with *S*.tm (post-stimulation) behave differently in regard to immune responses and infection control as compared to polarised macrophages subsequently exposed to bacteria (pre-stimulation). However, herein we uncover a novel role of IL-4 in the control of intracellular *Salmonella* infection in macrophages by modulation of L-arginine-dependent metabolic pathways.

## 2. Materials and Methods

### 2.1. Isolation of Bone-Marrow-Derived Macrophages

Bone-marrow-derived macrophages (BMDM) were isolated as previously described [34]. Briefly, the hind legs of female C57BL/6N, Tie2Cre^+/−^ARG1^fl/fl^ (KO) and Tie2Cre^−/−^ARG1^fl/fl^ (WT) [15] mice were removed. Bones were sterilised in 75% ethanol. Bone marrow was flushed out with ice-cold phosphate-buffered saline (PBS, Lonza, catalogue number: 17-515 F) supplemented with 1% penicillin/streptomycin (Lonza, catalogue number: DE17-602E). After erythrocyte lysis using M-lyse buffer concentrate (1×) (erythrocyte lysis kit, R&D, catalogue number: WL2000), cells were washed three times with PBS supplemented with 1% penicillin/streptomycin. For 5 days, cells were cultured in Dulbecco′s Modified Eagle′s Medium (DMEM, w: 1.0 g/L Glucose, *w*/*o*: L-Glutamine, w: Sodium pyruvate, w: 3.7 g/L NaHCO_3_, PanBiontech, catalogue number: P04-01500) supplemented with 10% FBS (PanBiontech, catalogue number: P30-3031), 1% penicillin/streptomycin and 1% L-glutamine (Lonza, catalogue number: BE17-605E) in the presence of 50 ng/mL recombinant murine macrophage colony-stimulating factor (MCSF; Peprotech, London, United Kingdom, catalogue number: 315-02) at 37 °C, 5% CO_2_ and saturated humidity in 15-cm cell culture dishes (Falcon, catalogue number: 352096). The medium was changed every second day. On day 6, cells were washed with PBS and scraped in a DMEM medium containing 10% FBS and 1% L-Glutamine. Afterwards, the cell number was determined using a LUNA-FL fluorescent and bright field automated cell counter (Biocat, catalogue number: L10001-LG). Cells were seeded at a density of 7 × 10^5^ per mL for RNA and CFU analysis in 6-well plates (Falcon, catalogue number: 353046), at a density of 5 × 10^6^ per mL for Western blot in 10 cm dishes (Falcon catalogue number: 351029) and 2 × 10^6^ per mL in 6 cm dishes (TTP, catalogue number: 93060) for FACS analysis.

### 2.2. Preparation of Salmonella Typhimurium

The pellet of *S*.tm (ATCC 14028) in the original vial was rehydrated in 1 mL of lysogeny broth (LB, Roth, catalogue number: X964.2) medium. Then, 10 µL of *S*.tm was pipetted into 10 mL of LB medium as a pre-culture and incubated overnight at 37 °C under constant shaking. The remaining 990 µL of rehydrated *S*.tm was centrifuged at 1920× *g* for 5 min. The pellet was resuspended in 1 mL of LB medium containing 30% glycerol. Aliquots of 100 µL per vial were prepared and frozen at −80 °C as stocks for future pre-cultures.

The following day, 50 µL of the pre-culture (stationary *S*.tm phase) was added to 10 mL of fresh LB medium and shaken at 37 °C for approximately 2 h, in order to reach an optical density of 0.5 at 600 nm (OD600). This value was set to ensure that the bacteria were in the logarithmic growth phase. *S*.tm was counted using a Casy Counting system (OMNI Life Science, Bremen, Germany catalogue number: TT-20A-2571) [35]. In addition, *Salmonella* Typhimurium (SL1344) expressing red fluorescent protein (RFP) were used for FACS analysis. They were kindly provided by Prof. Dr. Dirk Bumann (University of Basel, Switzerland).

### 2.3. Infection of Bone-Marrow-Derived Macrophages with Salmonella Enterica Serovar Typhimurium

**Figure 1:**
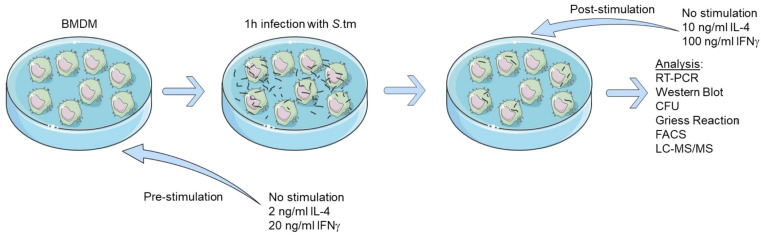
Schematic representation of in vitro stimulation and infection of BMDM with *S*.tm.

Cells were infected with *S*.tm as previously described [15,34]. In summary, cells were infected at a multiplicity of infection of 10 (MOI10). After 1 h of incubation, the medium containing non-phagocytosed *S*.tm was removed using a peristaltic pump. The cells were washed twice with PBS + 25 µg/mL gentamicin (Gibco, Vienna, Austria, catalogue number: 15750-037). After removing the extracellular *S*.tm with washing, PBS was removed using a peristaltic pump and 1 mL of DMEM medium supplemented with 10% FBS, 1% L-Glutamine and 25 µg/mL gentamicin. Cells were either left unstimulated or stimulated with 10 ng/mL IL-4 (Peprotech, London, united Kingdom, catalogue number: 214-14) or 100 ng/mL IFNγ (Peprotech, catalogue number: 315-05) for 4–24 h. For pre-polarisation of the macrophages, cytokines were added overnight before infection with *S*.tm at a concentration of 2 ng/mL IL-4 and 20 ng/mL IFNγ (Figure 1).

### 2.4. Arginine Free Media

A total of 50 mL of DMEM (w: 1.0 g/L Glucose, *w*/*o*: L-Glutamine, *w*/*o*: Amino acids, w: Sodium pyruvate, w: 3.7 g/L NaHCO_3_, PanBiontech, Aidenbach, Germany, catalogue number: P04-01507) was mixed with 500 µL L-Cystine (100 mM, Sigma-Aldrich, Vienna, Austria, catalogue number: C7602), 500 µL L-Leucine (100 mM, Sigma-Aldrich, catalogue number: L8912), 500 µL myo-Inositol (100 mM, Sigma-Aldrich, catalogue number: I7508), 500 µL L-methionine (100 mM, Sigma-Aldrich, catalogue number: M5308), 500 µL L-Glutamine (200 mM, Sigma-Aldrich, catalogue number: 59202C) and 500 µL HEPES (1M, Sigma-Aldrich, catalogue number: H3375). This medium was defined as L-arginine deficient medium. Cells were prepared as described and seeded into L-arginine deficient medium overnight. The following day, cells were infected with *S*.tm, and afterwards, cells were stimulated with 10 ng/mL IL-4 or 100 ng/mL IFNγ. After 4 and 24 h, CFU were analysed. Furthermore, L-arginine-deficient medium was supplemented with 500 µL of L-arginine (100 mM, Sigma, catalogue number: A6969), and the infection experiment was repeated.

### 2.5. Bacterial Growth Assay

A total of 10^4^ *S*.tm were incubated in 1 mL DMEM medium supplemented with 10% FBS and 1% L-Glutamine in a 14 mL round bottom tube (Falcon, catalogue number: 352059). *S*.tm were treated with 50 µM spermine (Sigma-Aldrich, catalogue number: S4264), 50 µM spermidine (Sigma-Aldrich, catalogue number: S0266), 50 µM putrescine (Sigma-Aldrich, catalogue number: P5780) or left untreated. Bacterial inoculates were incubated in a shaking platform at 37 °C for 14 h. Afterwards, bacterial numbers were determined by plating serial dilutions on LB agar plates. LB agar plates were incubated overnight at 37 °C. The following day, colonies were counted per hand, and CFU/mL were determined.

### 2.6. Analysis of Colony-Forming Units

The pathogen load of BMDM was analysed as described [15,34]. After infection with *S*.tm and stimulation for various time points, cells were washed three times with PBS to remove gentamicin. Then, cells were lysed with 0.5% Sodium-Deocycholic acid (Sigma-Aldrich, catalogue number: D6750-25G). Serial dilutions of the lysate were plated onto LB agar plates. Plates were incubated overnight at 37 °C. Colonies were counted per hand, and CFU/mL were determined.

### 2.7. The Multiplicity of Infection Test

Various MOIs were tested with cytokine stimulation. Therefore, cells were infected at a MOI 5, 1 and 0.5 for 1 h. Afterwards, the medium containing non-phagocytosed *S*.tm was removed using a peristaltic pump and the cell layer was washed twice with PBS containing 25 µg/mL gentamicin. Then, 1 mL of DMEM medium supplemented with 10% FBS, 1% L-Glutamine and 25 µg/mL gentamicin was added. Cells were either left unstimulated or stimulated with 10 ng/mL IL-4 or 100 ng/mL IFNγ for 30 min, 4 h or 24 h. Intracellular colony-forming units were determined.

### 2.8. RNA Isolation, Reverse Transcription and TaqMan Quantitative Real-Time PCR (qRT-PCR)

RNA from cells was isolated and reverse-transcribed as described [36]. Briefly, 500 µL of TRI Reagent^®^ (Sigma-Aldrich, catalogue number: T9424-200ML) was added to the cell layer, and RNA was gained according to the manufacturer’s protocol. M-MLV Reverse Transcriptase (Thermo Fisher Scientific, Vienna, Austria, catalogue number: 28025-021) was used for reverse transcription. Obtained cDNA was analysed for various gene expressions (Table 1) by quantitative real-time PCR using the CFX96 PCR system (BioRad, Hercules, CA, USA). The relative gene expression was calculated using the ∆∆Ct method.

### 2.9. Griess Reaction

Nitrite levels were measured as described [37]. Briefly, the supernatant of cell culture experiments was thawed and centrifuged at 300× *g* for 5 min. Then, 50 µL of the sample was pipetted into a 96-well plate (Life Science Products, catalogue number: 781602), and 100 µL of Griess–Ilosvay’s nitrite reagent (Merck, catalogue number: HX16599823) was added for 15 min. Afterwards, the plate was analysed at 546 nm using a PowerWave™ XS Microplate Reader (BioTek). The amount of nitrite was calculated based on a standard curve of a 1 M sodium nitrite (NaNO_2_, Sigma-Aldrich, catalogue number: 237213) solution.

### 2.10. Western Blot Analysis

Cells were lysed with cytoplasmic lysis buffer (3 g Tris (Roth catalogue number: 4855), 2.98 g KCl (Roth catalogue number: 6781), 10 mL Triton X100 (Roth catalogue number: 3051), adjust pH to 7.4, add 1000 mL Aqua bidest) for 15 min on ice. Samples were centrifuged for 5 min at 300× *g*. The supernatant was taken, and protein concentration was determined via Bradford analysis, following the manufacturer’s protocol using a Bio-Rad protein assay dye reagent concentrate (Biorad, catalogue number: 5000006). Immunoblotting was performed as described [38]. Protein levels of ARG1, iNOS, NRF2, SOD1, and β-ACTIN (Table 2) were detected using an ECL™ Prime Western Blotting System (Amersham Biosciences Europe GmbH, catalogue number: RPN2236) and analysed with a ChemiDoc Imaging system (Biorad).

### 2.11. FACS Analysis

FACS analysis of pre-stimulated or post-stimulated BMDM after 4 h and 24 h of infection was performed as described [34]. Briefly, cells were detached and pelleted. The pellet was resuspended in 50 µL surface staining mix (Table 3) for 10 min. After washing the pellet with 500 µL of PBS, cells were pelleted by centrifugation. The supernatant was aspirated, and cells were permeabilised and fixed with 50 µL Cytofix/Cytoperm buffer (BD Biosciences, catalogue number: 51-2090KZ) for 20 min at 4 °C. Then, 500 µL of Permwash buffer (BD Biosciences, catalogue number: 51-2091KZ) was added and samples were centrifuged. The supernatant was again aspirated, and the pellet was stained with 50 µL intracellular staining mix (Table 3) for 45 min at room temperature. After, 500 µL of Permwash buffer was added, and cells were pelleted. Pellet was resuspended in 200 µL of PBS. Samples were strained through a 40 µm sieve into a 96-well plate. Samples were analysed using a CytoFLEX S V4-B4-R2-I2 Flow Cytometer (13 detectors, 4 lasers, Beckman Coulter, catalogue number: C01161).

To determine differences between M1 and M2 polarisation states, BMDM were infected, stimulated and harvested as described. Cells were stained with 50 µL surface staining mix (Table 4). After incubation for 10 min at 4 °C, 500 µL of PBS was added to each sample and cells were pelleted. The supernatant was removed and the pellet was resuspended in 200 µL PBS. The samples were strained through a 40 µM sieve into a 96-well plate and analysed using a CytoFLEX S V4-B4-R2-I2 Flow Cytometer.

### 2.12. CellROX Measurement

Cells were prepared as described [39]. On the day of infection, 15 min before *S*.tm was added at a MOI 10, 0.5 µL of CellROX^TM^ Deep Red (Thermo Fisher, catalog number: C10422) was pipetted to each well. Cell culture dishes were incubated at 37 °C and 5% CO_2_ until infection. Cells were infected, and immediately after adding *S*.tm, the plate was placed in a pre-heated TecanSpark instrument to measure the production of reactive oxygen species at 625 nm (excitation) and 670 nm (emission). The fluorescent intensity of CellROX^TM^ was measured over the course of the infection period. Then, cells were washed twice with PBS containing 25 µg/mL gentamicin and new medium containing 25 µg/mL gentamicin and 0.5 µL/mL CellROX^TM^ was added. Fluorescent intensity was measured every hour. Differences between uninfected and infected BMDM, as well as post-stimulated BMDM, were analysed.

### 2.13. AlamarBlue Assay

Cellular cytotoxicity was measured by alamarBlue^®^ (BIORAD, catalogue number: BUF012A) according to the manufacturer’s protocol. Briefly, 2 × 10^4^ cells were seeded into a 96-well plate (Falcon, catalogue number: 353072) overnight in DMEM containing 10% FBS and 1% L-Glutamine. For pre-polarisation of the macrophages, cytokines were added overnight before infection with *S*.tm at a concentration of 2 ng/mL IL-4 and 20 ng/mL IFNγ or 10 ng/mL IL-4 and 100 ng/mL IFNγ. The next day, cells were infected with a MOI 10 of *S*.tm for 1 h. After 1 h of incubation, the medium containing non-phagocytosed *S*.tm was removed using a peristaltic pump. The cells were washed twice with PBS + 25 µg/mL gentamicin. After removing the extracellular *S*.tm with washing, PBS was removed using a peristaltic pump and 90 µL of DMEM medium supplemented with 10% FBS, 1% L-Glutamine and 25 µg/mL gentamicin was added. Cells were incubated for 24 h. The following day, 10 µL of alamarBlue^®^ reagent was added to the cells and 1 h of incubation was performed. Cellular cytotoxicity was determined measuring the fluorescent signal at an excitation/emission of 560 nm/590 nm with a TECAN Spark microplate reader.

### 2.14. LDH Assay

Cellular cytotoxicity was measured with a LDH cytotoxicity detection kit (Merck, catalogue number: 11644793001) according to the manufacturer’s protocol. Briefly, 2.5 × 10^5^ cells per mL were seeded into 12-well plates (Falcon, catalogue number: 353043) overnight in DMEM containing 1% L-Glutamine. The next day, cells were infected with a MOI 10 of *S*.tm for 1 h. After 1 h of incubation, the medium containing non-phagocytosed *S*.tm was removed using a peristaltic pump. The cells were washed twice with PBS + 25 µg/mL gentamicin. After removing the extracellular *S*.tm with washing, PBS was removed using a peristaltic pump and 1 mL of DMEM medium supplemented with 1% L-Glutamine and 25 µg/mL gentamicin was added. Cells were either left unstimulated or stimulated with 10 ng/mL IL-4 or 100 ng/mL IFNγ for 4 h. A total of 100 µL of the supernatant was transferred into a 96-well plate and 100 µL of the Dye Solution + Catalyst was added. The plate was incubated for 30 min in the dark. Absorbance was measured at 492 nm with a reference wavelength of 900 nm using a TECAN Spark microplate reader.

### 2.15. Metabolomics

For metabolic analysis, the cell layer was washed with ice-cold PBS three times. Afterwards, 1 mL of cold 100% methanol (MeOH, VWR, catalogue number: 20864.320) was added to each well. Cells were scraped, and the sample was transferred into a 1.5 mL tube (Eppendorf, catalogue number: 0030120.086). Cells were pelleted, and resuspended in 300 µL of a 1:1 methanol and water mixture. Cells were lysed by applying freeze–thaw cycles. Samples were placed into liquid nitrogen for 2 min and afterwards thawed at 37 °C for 2 min. This process was repeated four times. Samples were centrifuged at 16,000× *g* for 10 min, and the supernatants were transferred into new tubes. Determination of the levels of amino acids and biogenic amines induced by the different stimuli were accomplished with a targeted liquid chromatography tandem mass spectrometry (LC-MS/MS) method as described in the Supplementary Materials.

### 2.16. Statistical Analysis

Graphs are depicted as scatted dot plots with bars. Each symbol represents an individual value of one analysis. Data are presented as means ±  SEM. Statistical tests were performed with Graph Pad Prism 9. Significant differences between two groups were determined by a two-tailed Student’s *t*-test. For three or more groups, one-way ANOVA with Tukey post hoc test was applied. Three groups or more that also differed in another condition, for instance, pre-stimulation compared to post-stimulation, were analysed by two-way ANOVA with Tukey post hoc tests. *p*-values < 0.05 were considered significant.

## 3. Results

### 3.1. IL-4 Stimulation of Macrophages before or after Salmonella Infection Differently Affects Bacterial Multiplication

We were interested to evaluate how the polarisation state of bone-marrow-derived macrophages (BMDM) from C57BL/6N mice impact the control of infection with the intracellular bacterium *Salmonella* Typhimurium (*S*.tm). To this end, unpolarised BMDM or polarised BMDM (pre-treated with either 2 ng/mL IL-4 for M2 differentiation or 20 ng/mL IFNγ to induced M1 polarisation) were infected with *S*.tm for 1 h. Unpolarised BMDM were then stimulated (post-stimulation) with 10 ng/mL IL-4, 100 ng/mL IFNγ or left untreated. The phenotype of macrophages under these pre- and post-stimulation conditions depending on *S*.tm infection (uninfected, infected) and the time of stimulation (unstimulated, 4 h, 24 h) is shown in Supplementary Figure S1a–c. M1 macrophages are described as F4/80^+^CD11b^+^ CD80^high^CD206 ^low^, M2 macrophages are described as F4/80^+^CD11b^+^ CD80^low^CD206^high^.

**Figure 2:**
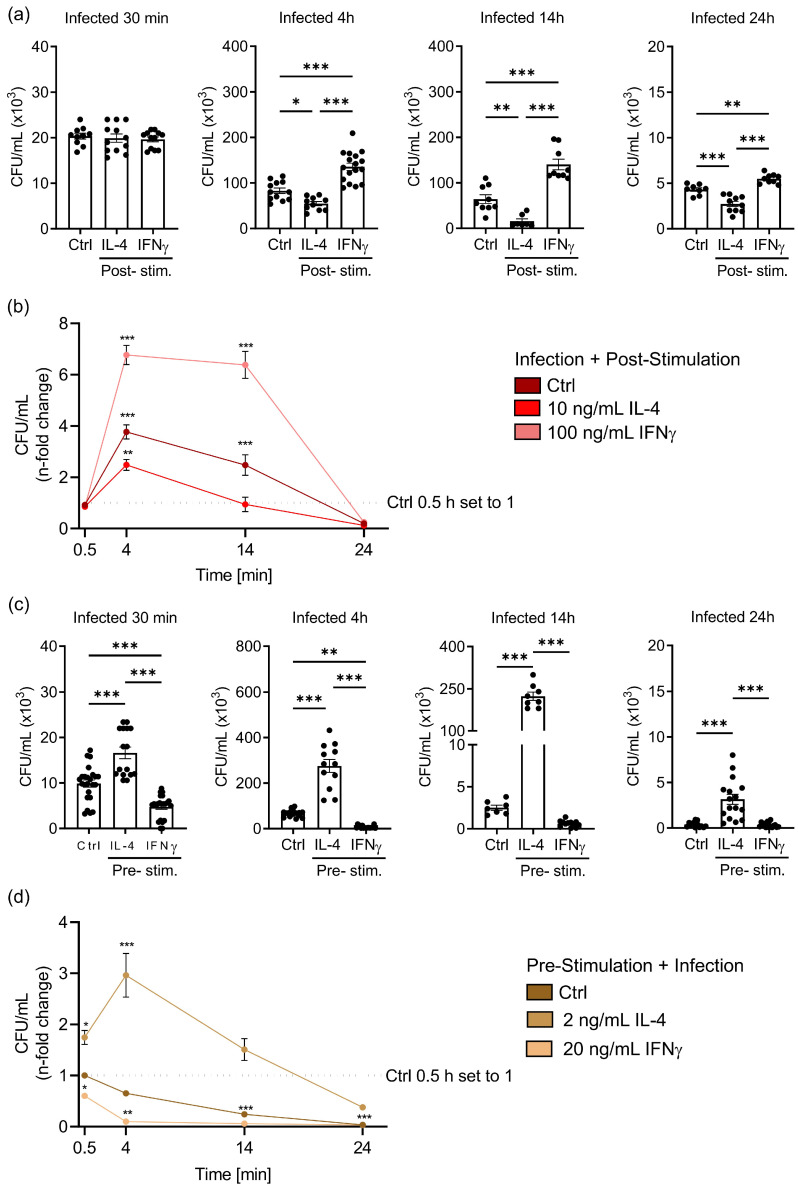
IL-4 stimulation of macrophages before or after infection determines the control of *Salmonella* infection. Unpolarised BMDM and pre-stimulated polarised BMDM (2 ng/mL IL-4 or 20 ng/mL IFNγ) were infected with *S*.tm for 1 h. Unpolarised cells were then stimulated (post-stimulation) with 10 ng/mL IL-4, 100 ng/mL IFNγ as indicated or left unstimulated. Differences between colony-forming units (CFU) were analysed. (**a**) Uptake of *S*.tm (Infected 30 min) and quantification of intracellular CFU in post-stimulated macrophages (Infected 4 h, 14 h, and 24 h). (**b**) Normalized intracellular CFU over time in post-stimulated BMDM. (**c**) Uptake of *S*.tm in pre-stimulated BMDM and killing capacity over the course of 24 h. (**d**) Normalized intracellular CFU over time in pre-stimulated BMDM. Statistical significance was determined by one-way ANOVA with Tukey post hoc test (a + c) and by two-way ANOVA with Tukey post hoc tests (b + d). * *p*-value < 0.05; ** *p*-value < 0.01; *** *p*-value < 0.001. Representative data (mean ± SEM) from three independent experiments performed in technical triplicates or quadruplicates are shown.

Next, *Salmonella* colony-forming units (CFU) were determined to analyse differences in bacterial killing capacity of post-stimulated macrophages over time (Figure 2a). No changes in *S*.tm uptake were observed after 30 min of infection when BMDM were stimulated with cytokines (post-infection). Importantly, over the course of 24 h, we observed a decrease of bacterial CFU in unpolarised BMDM, which were post-stimulated with IL-4 compared to non- cytokine treated infected BMDM (Ctrl). Interestingly, stimulation of infected but unpolarised BMDM with IFNγ led to an increase in CFU compared to Ctrl samples over the course of 24 h (Figure 2b).

Then, we primed BMDM with IL-4 or IFNγ overnight and then subsequently infected the cells with *S*.tm (pre-stimulation). To this end, we tested whether the concentrations of IL-4 and IFNγ we used in post-stimulation conditions were suitable for pre-stimulation experiments by measuring cytotoxicity with an AlamarBlue assay. We found an increase of cytotoxicity in pre-stimulation conditions when we used the same concentrations of IL-4 and IFNγ as in post-stimulation conditions. However, we found no differences in cytotoxicity in pre-stimulation when we reduced the cytokine concentrations to a fifth which, however, still resulted in polarisation of macrophages to either an M1 or M2 phenotype (Supplementary Figures S1a–c and S2).

Of interest, IL-4 priming in these pre-stimulation conditions resulted in higher bacterial numbers at 30 min after infection as compared to unpolarised macrophages (Ctrl), reflecting higher bacterial uptake. Follow up over time demonstrated significantly increased bacterial numbers with IL-4 polarised macrophages as compared to control and IFNγ pre-primed macrophages (Figure 2c). According to our hypothesis that the timing of stimulation with IL-4 or IFNγ is of importance for host defence against *S*.tm infection, BMDM pre-stimulated with IFNγ showed a reduction of bacterial numbers over time (Figure 2d).

From these data, we conclude that the timing of IL-4 and IFNγ stimulation to BMDM in relation to the initiation of infection opposingly affects host responses to intracellular *S*.tm, with the most striking observation that IL-4 stimulation of infected but unpolarised BMDM resulted in improved pathogen control by macrophages.

### 3.2. Priming of Macrophages before S.tm Infection Shows Differences in Arginase1 (ARG1) Regulation

**Figure 3:**
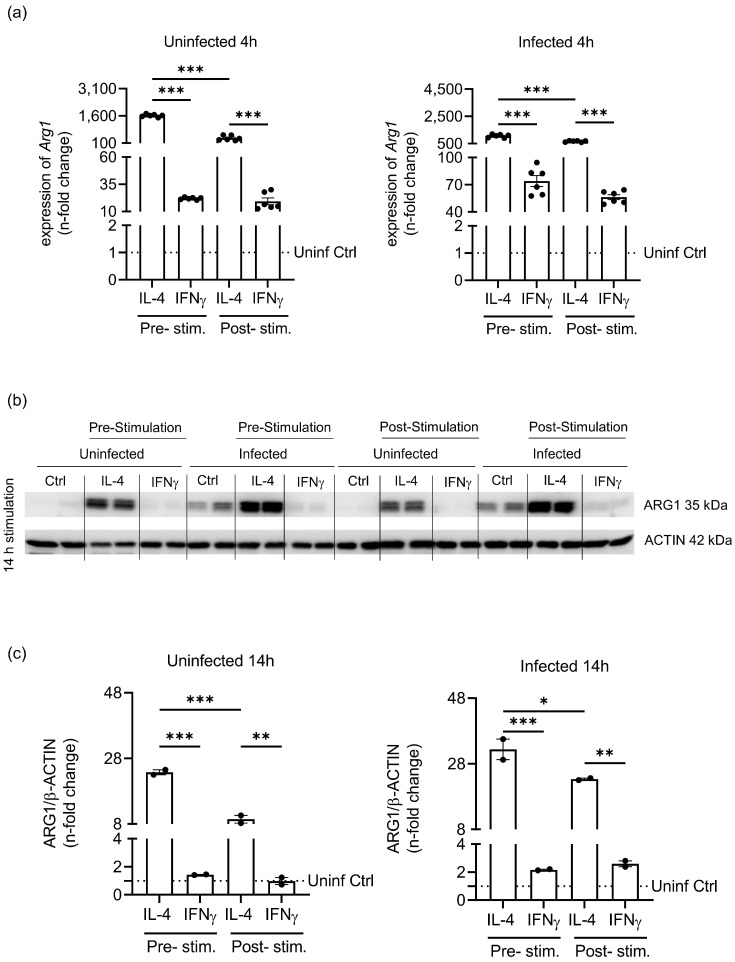
Priming of macrophages before *S*.tm infection shows differences in ARG1 regulation. Unpolarised BMDM and pre-stimulated polarised BMDM (2 ng/mL IL-4 or 20 ng/mL IFNγ) were infected with *S*.tm for 1 h. Unpolarised cells were then stimulated (post-stimulation) with 10 ng/mL IL-4, 100 ng/mL IFNγ or left unstimulated for 4 h or 14 h. ARG1 mRNA and protein levels in pre- and post-stimulation conditions were analysed in uninfected and infected macrophages. (**a**) *Arg1* gene expression depending on stimulation (IL-4 or IFNγ) and infection. *Arg1* transcript levels were determined by quantitative real-time PCR and normalized to Hypoxanthine phosphoribosyltransferase (*Hprt*) mRNA levels using the ΔΔCT method. (**b**) Western blot analysis of ARG1 and β-ACTIN. (**c**) Densidometrical quantification of immunoblotting results relative to β-ACTIN expression. Statistical significance was determined by one-way ANOVA with Tukey post hoc test (a + c). * *p*-value < 0.05; ** *p*-value < 0.01; *** *p*-value < 0.001. Representative data from three independent experiments (mean ± SEM) performed in technical duplicates are shown. Data were normalised to the unstimulated, uninfected Ctrl.

We then investigated the mechanisms underlying the differences in infection control of pre-stimulated and post-stimulated macrophages. To this end we analysed the expression of the two main effector enzymes of macrophages, iNOS and ARG1, at the mRNA and protein level. We found that infection of macrophages with *S*.tm resulted in increased mRNA and protein expression of *Arg1* and *iNos* in F4/80^+^CD11b^+^ macrophages (Supplementary Figure S3a–c). Importantly, priming of macrophages with IL-4 (pre-stimulation) increased *Arg1* expression compared to BMDM stimulated with IL-4 after establishment of the infection. Macrophages pre-primed with IFNγ showed slightly higher levels of *Arg1* mRNA upon infection compared to unpolarised infected macrophages treated with IFNγ after onset of infection. However, compared to IL-4 treated samples, gene expression of *Arg1* was significantly reduced in all conditions with IFNγ stimulation (Figure 3a). The slight induction of *Arg1* mRNA expression in uninfected IFNγ-treated samples when compared to the uninfected controls was not translated into increased protein levels (Figure 3a,b). Essentially, protein levels of ARG1 correlated with mRNA expression as IL-4 stimulation increased ARG1 protein levels whereas IFNγ stimulation reduced it. Accordingly, in infected macrophages higher ARG1 levels were detected (Figure 3b,c).

**Figure 4:**
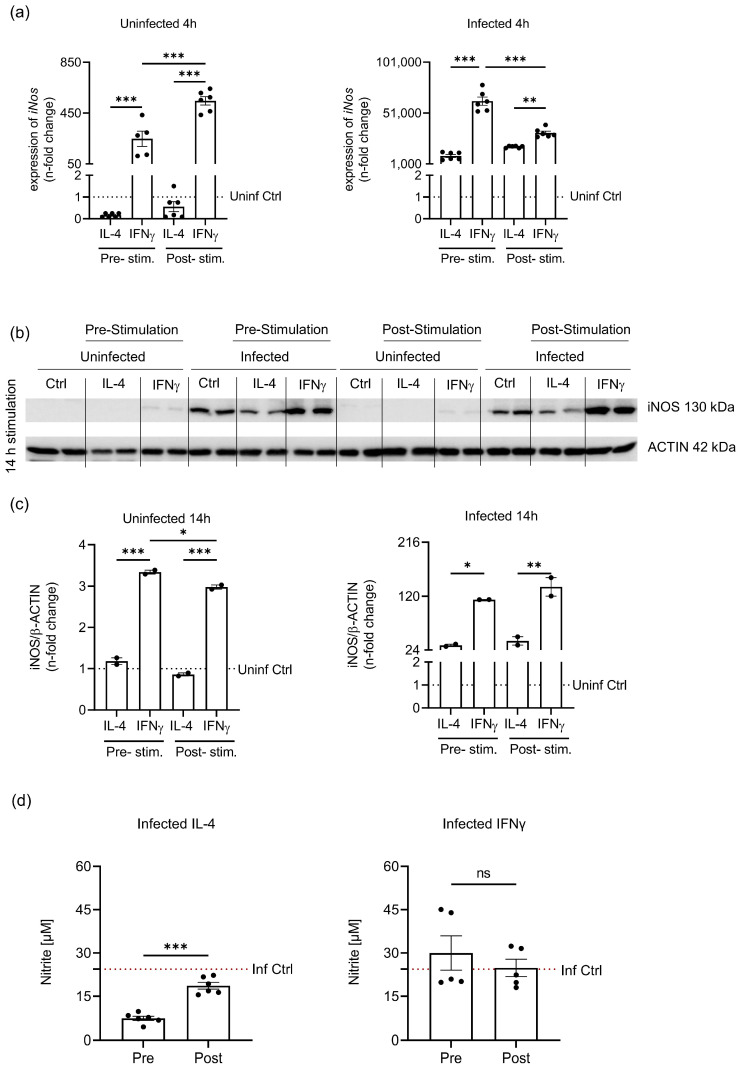
Priming of macrophages before *S*.tm infection shows differences in iNOS regulation. Unpolarised BMDM and pre-stimulated polarised BMDM (2 ng/mL IL-4 or 20 ng/mL IFNγ) were infected with *S*.tm for 1 h. Unpolarised cells were then stimulated (post-stimulation) with 10 ng/mL IL-4, 100 ng/mL IFNγ or left unstimulated for 4 h or 14 h. *iNOS* mRNA and protein levels in pre- and post-stimulation conditions were analysed in uninfected and infected macrophages. (**a**) *iNos* gene expression depending on stimulation (IL-4 or IFNγ) and infection. *iNos* transcript levels were determined by quantitative real-time PCR and normalised to Hypoxanthine phosphoribosyltransferase (*Hprt*) mRNA levels using the ΔΔCT method. (**b**) Western blot analysis of iNOS and β-ACTIN. (**c**) Densidometrical quantification of immunoblotting results relative to β-ACTIN expression. (**d**) Nitrite levels of pre- and post-stimulated samples after 14 h determined via Griess reaction. Statistical significance was determined by one-way ANOVA with Tukey post hoc test (a + c). * *p*-value < 0.05; ** *p*-value < 0.01; *** *p*-value < 0.001; ns = not statistically significant. Representative data from three independent experiments (mean ± SEM) performed in technical duplicates are shown. Data were normalised to the unstimulated uninfected Ctrl.

iNOS mRNA and protein expression increased after IFNγ pre- and post-stimulation conditions upon infection with *S*.tm as compared to the uninfected control (Figure 4a–c), whereas IL-4 under pre- and post-stimulation conditions even reduced it below control conditions. This was true for infected and uninfected macrophages (Figure 4, Supplementary Figure S3a,b). Accordingly, nitrite levels with IL-4 pre- and post-stimulation were lower than in infected controls. IFNγ stimulation led to a slight increase of nitrite levels; however, no significant differences could be detected between IFNγ pre- and post-stimulation conditions (Figure 4d).

Taken together, these data indicate that the time point when macrophages are stimulated by cytokines in the course of an infection with *S*.tm accompanied by the modification of ARG1 and iNOS expressions is of utmost importance for controlling an infection with *Salmonella*.

### 3.3. Pre- or Post-Stimulation of Macrophages with IL-4 and IFNγ Impacts the Amount of S.tm Phagocytosed by F4/80+CD11b+ Macrophages

We next tested whether the alterations of *S*.tm CFU in pre- or post-stimulated BMDM correlated with the percentage of macrophages containing *Salmonella*. To this end, we repeated the pre- and post-stimulation experiments using a *Salmonella* strain which expresses red-fluorescent protein (RFP). Macrophages were gated according to Supplementary Figure S4.

**Figure 5:**
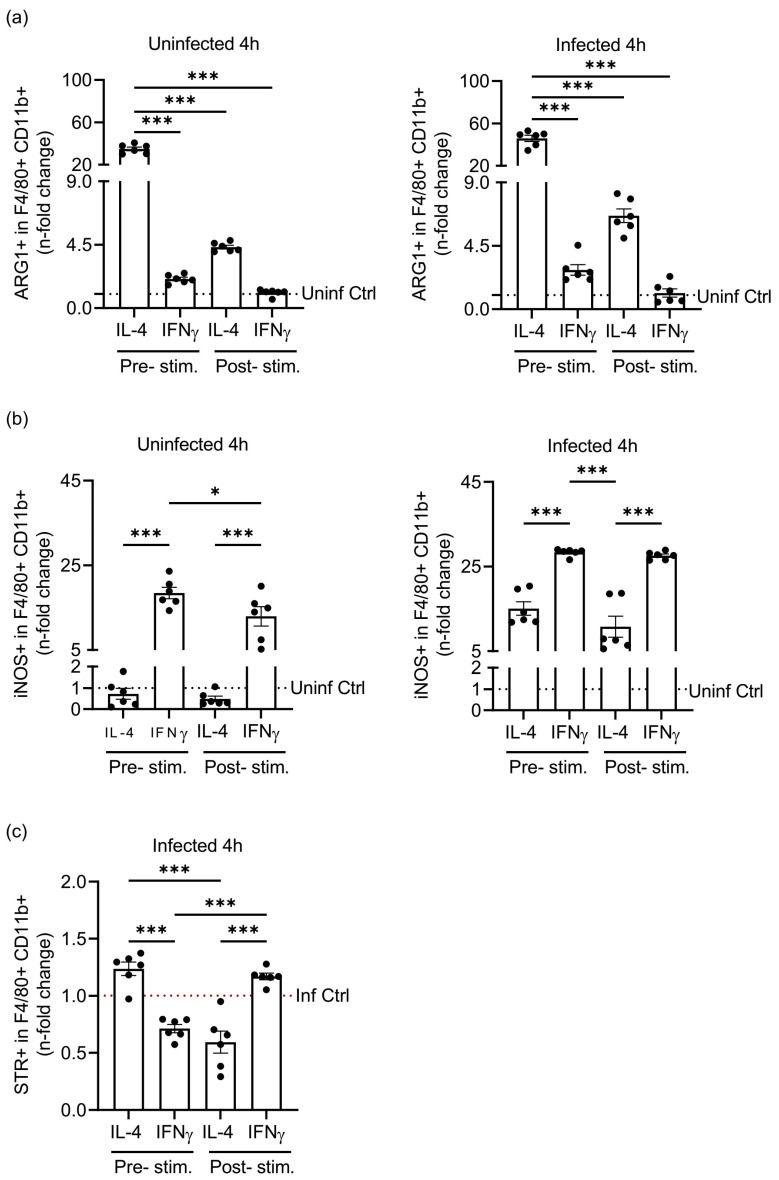
Pre- or post-stimulation of macrophages with IL-4 and IFNγ impacts the amount of *S*.tm phagocytosed by F4/80^+^CD11b^+^ macrophages. Unpolarised BMDM and pre-stimulated polarised BMDM (2 ng/mL IL-4 or 20 ng/mL IFNγ) were infected with *S*.tm expressing RFP (STR) for 1 h. Unpolarised cells were then stimulated (post-stimulation) with 10 ng/mL IL-4, 100 ng/mL IFNγ or left unstimulated for 4 h. Percentages of ARG1, iNOS and STR in macrophages were analysed via flow cytometry. (**a**) Percentage of ARG1-positive cells in F4/80^+^CD11b^+^ cells in pre-stimulated compared to post-stimulated BMDM. Data shown for uninfected (left) and infected (right) cells. (**b**) Percentage of iNOS positive cells in F4/80^+^CD11b^+^ in pre-stimulated compared to post-stimulated BMDM. Data shown for uninfected (left) and infected (right) cells. (**c**) Percentage of STR positive cells in F4/80^+^CD11b^+^ cells in infected pre-stimulated compared to post-stimulated BMDM. Statistical significance was determined by one-way ANOVA with Tukey post hoc test. * *p*-value < 0.05; *** *p*-value < 0.001. Representative data (mean ± SEM) from three independent experiments with two technical replications are shown. Data were normalised to the unstimulated uninfected Ctrl or, as indicated, to the unstimulated infected Ctrl.

According to mRNA expression and protein levels, we found that upon IL-4 stimulation of F4/80^+^CD11b^+^ macrophages, ARG1 levels were increased, and iNOS expression was reduced. However, under pre-stimulation conditions, IL-4 treatment resulted in significantly more ARG1^+^ cells when compared to post-stimulation conditions. Infection with *S*.tm further increased ARG1 expression in F4/80^+^CD11b^+^ macrophages. This was seen for all time points analysed (Figure 5a, Supplementary Figure S5a). When compared to the uninfected unstimulated control (Supplementary Figure S3c), pre- and post-stimulation with IFNγ showed a minimal increase of ARG1^+^ macrophages; however, in infected samples IFNγ stimulation decreased the amount of ARG1^+^ cells compared to unstimulated infected controls (Figure 5a left, Supplemental Figure S5a right, Supplementary Figure S3c).

In regard to iNOS expression, however, IFNγ pre- and post-stimulation showed opposite results. In uninfected cells, post-stimulation with IFNγ reduced the levels of iNOS^+^ cells in F4/80^+^CD11b^+^ macrophages compared to pre-stimulation conditions. Infection with *S*.tm further increased the number of iNOS^+^ cells (Figure 5b, Supplementary Figure S5b). On the contrary, IL-4 stimulation of uninfected BMDM only marginally influenced the expression of iNOS by F4/80^+^CD11b^+^ macrophages, and those levels remained lower following *S*.tm infection compared to IFNγ pre- and post-stimulation (Figure 5b, Supplementary Figure S5b).

Furthermore, we confirmed that the alterations in CFU levels in pre- or post-stimulation conditions (Figure 2) correlated with the percentage of STR-positive cells in pre- compared to post-stimulated macrophages after 4 h and 24 h of infection (Figure 5c, Supplemental Figure S5c).

### 3.4. Different Multiplicities of Infection Do Not Alter the Effects of IL-4 and IFNγ Stimulation on the Growth of S.tm in Infected Macrophages

To exclude the possibility that the IL-4 post-stimulation-dependent reductions in *S*.tm CFU were dependent on the ratio of bacteria to macrophages, we investigated these observations at a multiplicity of infection (MOI) to 0.1, 1 and 5 and analysed *S*.tm CFU after 0.5 h, 4 h and 24 h of infection.

**Figure 6:**
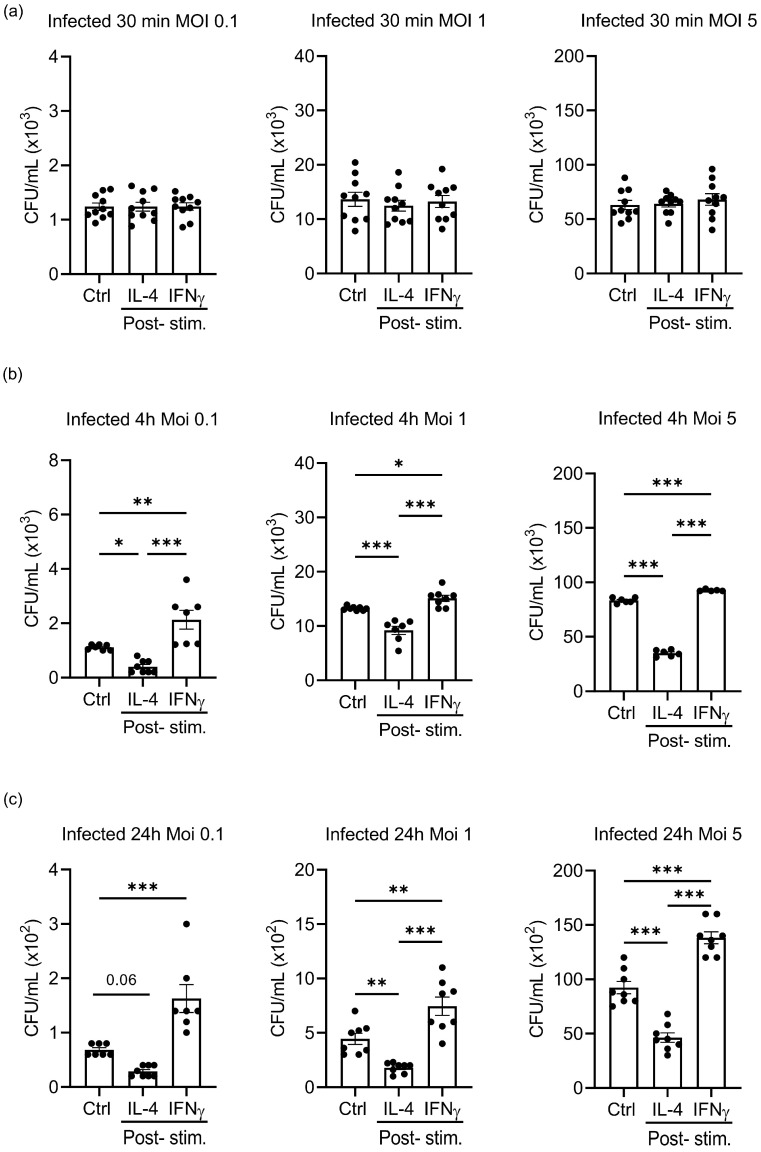
Different multiplicities of infection do not alter the effects of IL-4 and IFNγ stimulation on the growth of *S*.tm in macrophages. Unpolarised BMDM were infected with *S*.tm for 1 h. Afterwards, cells were stimulated (post-stimulation) with 10 ng/mL IL-4, 100 ng/mL IFNγ or left unstimulated as indicated. (**a**) CFU analysis after 0.5 h (**a**), 4 h (**b**) and 24 h (**c**) of infection with several multiplicities of infection (MOI) of *S*.tm in post-stimulated BMDM. Statistical significance was determined by one-way ANOVA with Tukey post hoc test: * *p*-value < 0.05; ** *p*-value < 0.01; *** *p*-value < 0.001. Representative data (mean ± SEM) from three independent experiments with 2–3 technical replications are shown.

After 30 min of post-stimulation, which is assessed to reflect the ingestion of bacteria by macrophages, no differences in *S*.tm CFU were detected with different cytokine stimulations at MOI of 0.1, 1, or 5. (Figure 6a).

However, after 4 h and 24 h of infection, independent of the MOIs we used, we confirmed the significant reduction of *S*.tm CFU in IL-4 post-stimulation conditions in a similar fashion as seen in infected macrophages at a MOI 10 (Figure 6b,c). Similarly, IFNγ treatment of unpolarised macrophages after establishment of infection led to higher *S*.tm CFU in BMDM as compared to IL-4 stimulation.

Thus, the IL-4-mediated control of bacterial multiplication does not depend on the ratio of bacteria to macrophages.

### 3.5. IL-4 Stimulation of Unpolarised S.tm-Infected BMDM Does Not Affect Cellular Viability, Radical Formation and Expression of Radical Detoxifying Enzymes

**Figure 7:**
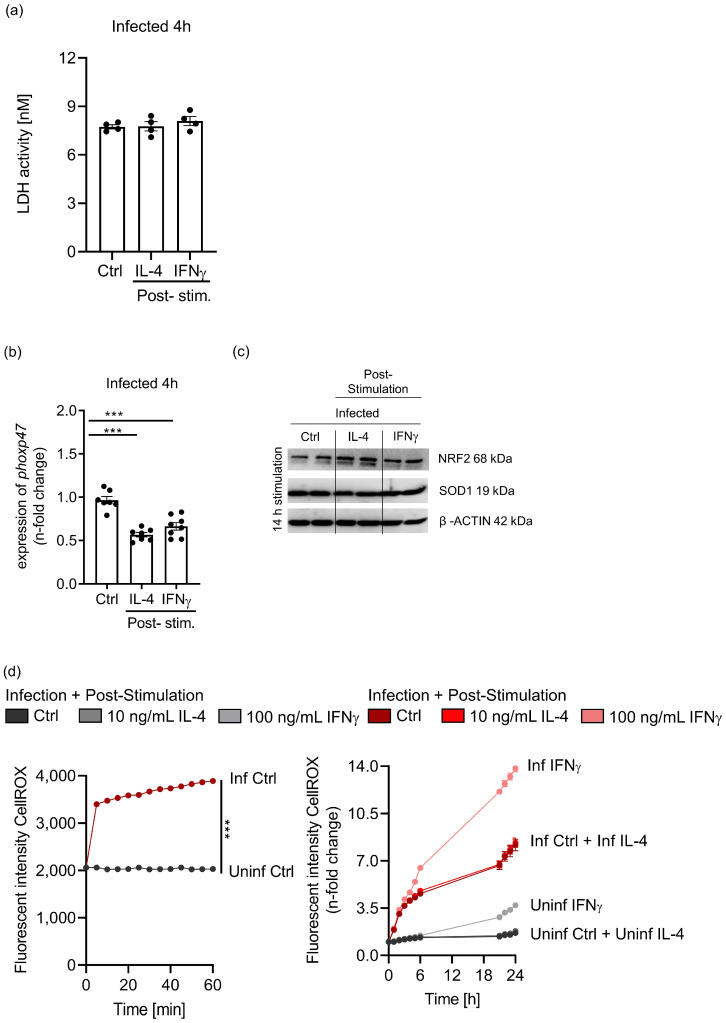
IL-4 stimulation of unpolarised *S*.tm-infected BMDM does not affect cellular viability, radical formation and expression of radical detoxifying enzymes. Unpolarised BMDM were infected with *S*.tm for 1 h. Afterwards, cells were stimulated (post-stimulation) with 10 ng/mL IL-4, 100 ng/mL IFNγ or left unstimulated as indicated. (**a**) Determination of cell viability by analysing LDH activity after 4 h post-stimulation. (**b**) *Phoxp47* transcript levels due to infection and 4 h post-stimulation were determined by quantitative real-time PCR and normalized to Hypoxanthine phosphoribosyltransferase (*Hprt*) mRNA levels using the ΔΔCT method. Data were normalised to the infected Ctrl. (**c**) Western blot analysis of SOD1, NRF2 and β-ACTIN expression after 14 h. (**d**) Quantification of ROS formation using the CellROX^TM^ reagent, during a 1 h infection period (left) and during a gentamicin neutralisation assay and post-stimulation over the course of 24 h. Time course data were normalised to the levels of the 30 min unstimulated uninfected Ctrl. Statistical significance was determined by one-way ANOVA with Tukey post hoc test for three groups and two-way ANOVA with Tukey post hoc tests for more groups: *** *p*-value < 0.001. Representative data (mean ± SEM) from two to three independent experiments with two technical replications are shown.

To further determine whether IL-4 or IFNγ stimulation could impact BMDM viability during the course of an infection with *S*.tm, we measured LDH levels, which showed no significant differences between the different treatment groups, thereby excluding an influence of IL-4 or IFNγ stimulation on cellular viability (Figure 7a).

Next we tested whether regulation of cytoprotective proteins or levels of ROS production could be responsible for the increased infection control by IL-4. We quantified the expression of *Phoxp47*, a central component of NADPH oxidase, which is an oxygen radical-forming enzyme involved in immune control of *Salmonella* infection by macrophages [40]. We observed a reduction of *Phoxp47* gene expression in both IL-4 and IFNγ post-stimulated infected BMDM compared to infected, non-cytokine stimulated BMDM (Figure 7b), whereas no significant difference became evident when comparing treatment with IL-4 and IFNγ.

Next we analysed protein expression of nuclear factor erythroid 2–related factor 2 (NRF2) and superoxide dismutase [Cu-Zn] (SOD1), both important in regulating ROS-mediated stress responses in macrophages (Figure 7c), which demonstrated no differences between the various treatment regimens. This indicates that neither oxygen radical formation by the NADPH oxidase, nor ROS-mediated stress response regulation by NRF2 or SOD1, appears to be responsible for the decreased *S*.tm survival in infected BMDM treated with IL-4.

To study whether increased ROS levels were responsible for the decreased *S*.tm survival in infected BMDM treated with IL-4, we quantified ROS formation using the CellROX^TM^ reagent. As a result of oxidation, this reagent exhibits strong fluorescence and persists within the cells. Increased fluorescent intensity of CellROX^TM^ was measured immediately after adding *S*.tm to the BMDM compared to uninfected macrophages (Figure 7d, left). As expected, treatment of BMDM with IFNγ led to an increase of measurable CellROX^TM^ fluorescent intensity whereby IFNγ addition to infected BMDM produced the highest amounts of ROS when measured over time (Figure 7d, right). Importantly, BMDM infected with *S*.tm and post-stimulated with IL-4 showed similar CellROX^TM^ levels compared to infected control samples.

In summary, reduced CFU levels in infected and subsequently IL-4 stimulated macrophages were neither due to changes in cellular viability, upregulation of cytoprotective proteins nor enhanced ROS production.

### 3.6. IL-4 Treatment of S.tm.-Infected BMDM Affects L-arginine-Driven Metabolic Pathways Causing Accumulaiton of Putrescine, Spermidine, and Spermine

**Figure 8:**
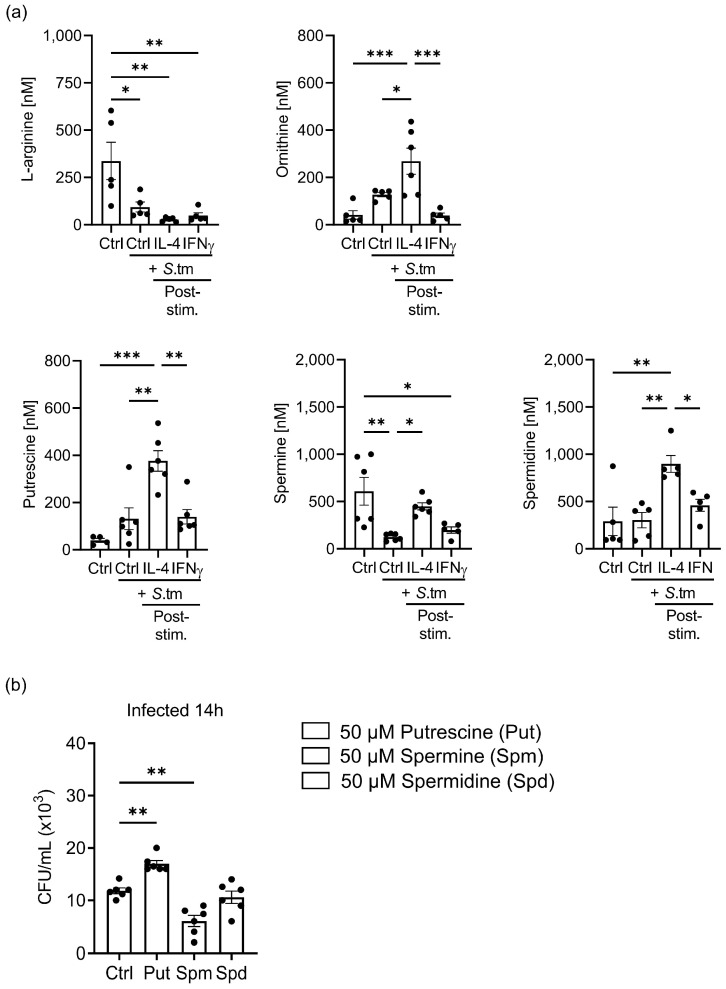
IL-4 stimulation of unpolarised macrophages after infection with *S*.tm alters L-arginine metabolic pathways with direct impact on the growth of *Salmonella*. Unpolarised BMDM were infected with *S*.tm for 1 h. Afterwards, cells were stimulated (post-stimulation) with 10 ng/mL IL-4, 100 ng/mL IFNγ or left unstimulated as indicated. (**a**) Concentrations of L-arginine and related metabolites determined by LC-MS/MS in 24 h post-stimulated BMDM cell lysates. (**b**) Growth of *S*.tm after 14 h in media supplemented with either 50 µM spermine, 50 µM spermidine or 50 µM putrescine. Statistical significance was determined by one-way ANOVA with Tukey post hoc test. * *p*-value < 0.05; ** *p*-value < 0.01; *** *p*-value < 0.001. Representative data (mean ± SEM) of three independent experiments with 2–4 technical replications are shown.

Both enzymes, iNOS and ARG1, need L-arginine as a substrate and therefore are competing for it. Thus, we measured the alterations of L-arginine and its metabolites in uninfected and infected BMDM cell lysates upon different treatment conditions (Figure 8a). *Salmonella* infection led to decreased L-arginine levels. Stimulation of *Salmonella*-infected BMDM led to increased levels of ornithine and its catabolites putrescine, spermidine, and spermine. Post-stimulation with IFNγ, on the contrary, reduced the formation of those metabolites originating from the arginase pathway. We then examined whether these metabolites of the arginase pathway could possibly influence the growth of *S*.tm. While putrescine supplementation to the growth medium led to an increase of *Salmonella* proliferation when compared to un-supplemented control samples, spermine supplementation significantly decreased *S*.tm numbers (Figure 8b).

**Figure 9:**
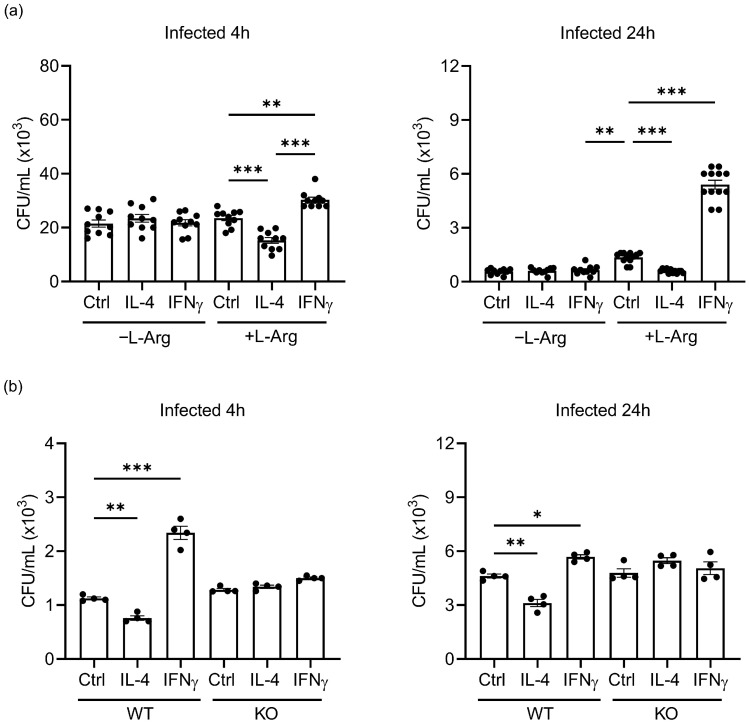
IL-4 exerts its inhibitory effect on *S*.tm proliferation via L-arginine-mediated metabolic pathways. Effects of L-arginine depletion and deletion of ARG1 on *S*.tm survival in macrophages was analysed. BMDM generated from C57BL/6N mice were cultured in L-arginine-free medium. Moreover, BMDM of Tie2Cre^+/−^ARG1^fl/fl^ (KO) and Tie2Cre^−/−^ARG1^fl/fl^ (WT) were generated. Macrophages were infected with *S*.tm and stimulated (post-stimulation) with 10 ng/mL IL-4, 100 ng/mL IFNγ or left unstimulated as indicated. (**a**) Effect of arginine-depleted medium (− L-Arg) on survival of *S*.tm in BMDM compared to *S*.tm survival in medium supplemented with L-arginine (+ L-Arg). (**b**) *S*.tm CFU in Tie2Cre^−/−^ARG1^fl/fl^ (WT) BMDM compared to Tie2Cre^+/−^ARG1^fl/fl^ (KO) BMDM after post-stimulation as indicated. Statistical significance was determined by one-way ANOVA with Tukey post hoc test: * *p*-value < 0.05; ** *p*-value < 0.01; *** *p*-value < 0.0001. Representative data (mean ± SEM) of two to three independent experiments with 2–4 technical replications are shown.

To further explore the influence of IL-4 post-stimulation on the L-arginine pathway and therefore on *S*.tm proliferation in macrophages, we infected IL-4 or IFNγ post-stimulated BMDM in L-arginine-depleted medium and studied the effects on the course of infection under these conditions and upon supplementation of L-arginine after establishment of infection and cytokine treatment. Under L-arginine-depleting conditions, no differences in *S*.tm CFU could be seen when infected BMDM, without cytokine stimulation or subsequent IL-4 or IFNγ (post-stimulation), were compared after 4 h and 24 h of infection. However, supplementation of L-arginine could restore the effects of IL-4 stimulation toward inhibition of *S*.tm proliferation and the increase of *S*.tm numbers in IFNγ post-stimulated macrophages after 4 h and 24 h (Figure 9a). This indicated that IL-4-mediated induction of ARG1 and subsequent metabolic breakdown of L-arginine to the metabolites spermine and spermidine are responsible for the protective effect of IL-4 on control of *S*.tm. proliferation in infected BMDM. To further confirm this mechanism, we generated BMDM from Tie2Cre^+/−^ARG1^fl/fl^ knockout (KO) mice and Tie2Cre^−/−^ARG1^fl/fl^ wildtype littermates (WT). Cells were infected with *S*.tm and subsequently stimulated with IL-4 or IFNγ. As compared to cells expressing ARG1, the deletion of ARG1 resulted in disappearance of the differences in *S*.tm control by IL-4 and IFNγ (Figure 9b).

L-arginine-derived metabolites originating from breakdown of the amino acid by ARG1 and subsequent enzymatic degradations thus appear to be central for IL-4-induced reduction of *S*.tm in unpolarised infected BMDM.

## 4. Discussion

Here we report a novel role of IL-4 for the control of intracellular *Salmonella* infection in macrophages by modulating L-arginine-dependent metabolic pathways. Importantly, we found that unpolarised macrophages which are exposed to IL-4 after establishment of infection with *S*.tm are able to better control bacterial multiplication.

This is in line with earlier studies indicating that differences in the macrophage polarisation state determine antimicrobial host responses and therefore the susceptibility and outcome of infectious diseases with various pathogens [3,4,5,6]. This involves the induction of the NO-generating enzyme iNOS, efficiently induced by IFNγ, and the L-arginine-depleting enzyme arginase 1 (ARG1), stimulated by IL-4 [4,5,6,8,41,42].

In our model, unpolarised BMDM or BMDM polarised with IL-4 or IFNγ were infected with *S*.tm. Interestingly, IL-4 stimulation after infection (post-stimulation) markedly reduced intracellular bacterial numbers over a period of up to 24 h. In contrast, stimulation of unpolarised macrophages with IFNγ after establishment of infection significantly increased *Salmonella* CFU over time. These findings demonstrate that the macrophage polarisation stage and timing of cytokine stimulation critically affects microbicidal activity of macrophages.

During the initial phase of systemic *Salmonella* infection, a pro-inflammatory immune response is initiated, mainly driven by pro-inflammatory (M1) macrophages and the T cell and NK cell derived cytokine IFNγ. It was shown recently that in later stages of infection *Salmonella* preferentially associates with anti-inflammatory (M2) macrophages, which may be more permissive for intracellular bacteria [31,32,33]. Accordingly, if bacteria were administered to macrophages polarised with IFNγ to the M1 phenotype, intracellular CFU of *Salmonella* significantly decreased. Compared to this, pre-stimulation with IL-4 leading to an anti-inflammatory (M2) phenotype prior to infection increased intracellular bacterial numbers.

In support of our findings, a recent study demonstrated that pre-treatment of peritoneal macrophages with IL-4 enhances the expression of pro-inflammatory genes (TNF-α, IL-1α, KC, MIP-2) following an inflammatory stimulus. This could be explained by the expression of the IL-12p40 gene, which is essential for the production of IFNγ, and which is inhibited by IL-4 pre-treatment [43]. This well-known ability of IL-4 to suppress an efficient production of IFNγ is most likely reflected in our pre-stimulation model and therefore responsible for the increase of CFU under IL-4 pre-stimulated conditions, resulting in polarisation of macrophages to the M2 phenotype. Moreover, it was shown that mortality of mice treated with a human–mouse chimera of IL-12 was significantly increased after challenge with Gram-negative bacteria E.coli and that this was directly related to IFNγ induction [44]. Recently, another study demonstrated that in a mouse model of acute lung injury, neutralization of IFNγ led to a significant reduction of the disease outcome and improved the outcome of mortality [45]. Furthermore, in moderate to severe COVID-19 infections, a sustained increase of IFNγ levels was related to increased mortality [46]. These conditions resemble our CFU results of IFNγ post-stimulated BMDM, as we observed an impaired infection control by these macrophages.

Mechanistically, for host resistance to infection by intracellular microbes, various L-arginine catabolising pathways are crucial. Accordingly, our data showed that L-arginine levels were significantly decreased in all infected groups. However, in IL-4 post-stimulation conditions, a rise in polyamine synthesis was observed. This is in line with earlier studies indicating that IL-4 promotes macrophages to convert L-arginine to ornithine through ARG1 and that ornithine is further processed into the polyamines putrescine, spermine, and spermidine [47]. Albeit a recent study describing that the signalling via IL-4 receptor-α is crucial for the lifespan of monocytes [48], we could not see any effects of IL-4 stimulation on cellular viability, radical formation, or expression of radical detoxifying enzymes in our in vitro model. This indicates that indeed the L-arginine metabolism is responsible for microbicidal macrophage activity and that IL-4 per se has no toxic effects during infection.

Our findings raise the question how the increase of polyamines impact on the growth of *S*.tm. According to our data, bacterial growth was significantly reduced when *S*.tm grew in media supplemented with spermine. Accordingly, a recently published study identified spermine as an important DNA-condensing factor associated with self-non-self recognition. Thereby, depletion of spermine resulted in attenuated antiviral and anticancer immunity [49]. This is accompanied by the clinical observation that inhibition of polyamines had no benefit for cancer patients [50]. Thus, it is tempting to assume that the increase in polyamines in unpolarised BMDM stimulated with IL-4 after infection with *S*.tm is primarily driven by the metabolisation of L-arginine by IL-4-dependent ARG1. In our model, this leads to a better infection control which is reflected by a decrease of intracellular *Salmonella* CFU. This hypothesis is further strengthened by the finding that the depletion of L-arginine from cell culture media resulted in unchanged *S*.tm levels, even when stimulated with IL-4 after infection.

Our findings further suggest that *S*.tm multiplication is impaired due to the reduction of L-arginine as an energy source. The arginine pool of the host can be utilized by intracellular pathogens, leading to the expression of a number of genes involved in pathogenicity. This is in line with recent discoveries highlighting the importance of L-arginine as an energy source [51,52].

In summary, our study demonstrates that the induction of a metabolic reprogramming of the L-arginine pathway by IL-4 reduces the proliferation of *S*.tm in unpolarised macrophages. Furthermore, it was shown that this depends on the differentiation status of macrophages, as only unpolarised macrophages which were supplemented with IL-4 show a better infection control by impacting the L-arginine pathway.

Even though the polarisation of macrophages by cytokines is an attractive model to study infectious and inflammatory diseases in vitro and ex vivo, a better understanding of macrophage differentiation and effects of cytokines on host responses to infection in vivo are necessary.

## Figures and Tables

**Table 1:** Sequences of primers and TaqMan probes.

Murine Gene	Forward Primer 5′-3′	Reverse Primer 5′-3′	Probe
*Hprt*	GACCGGTCCCGTCATGC	TCATAACCTGGTTCATCATCGC	ACCCGCAGTCCCAGCGTCGTC
*Arg1*	AACACGGCAGTGGCTTTAAC	GAGGAGAAGGCGTTTGCTTA	TGGCTTATGGTTACCCTCCCGTTG
*iNos*	CAGCTGGGCTGTACAAACCTT	CATTGGAAGTGAAGCGTTTCG	CGGGCAGCCTGTGAGACCTTTGA
*Phoxp47*	GAGGCGGAGGATCCGG	TCTTCAACAGCAGCGTACGC	CAACTACGCAGGTGAACCGTATGTAACCATCA

**Table 2:** Antibodies used for immunoblotting.

Detected Protein	Antibody Dilution	Clone Number	Commercial Source	Catalogue Number
ARG1	1:2000	Polyclonal	Novusbio	NBP1-32731
iNOS	1:1000	Polyclonal	Abcam	ab15323
SOD1	1:1000	Polyclonal	Enzo	ADI-SOD-100
NRF2	1:1000	D1Z9C	CellSignalling	12721
β-ACTIN	1:500	Polyclonal	Sigma-Aldrich	A 2066

**Table 3:** FACS antibodies for macrophage staining.

Antibody against	Conjugated Fluorochrome	Clone Number	Commercial Source	Catalogue Number
Surface:				
CD3	FITC	17.A2	ImmunoTools	100204
CD19	FITC	PeCa1	ImmunoTools	22220193S
CD49b	FITC	HMa2	BD Biosciences	103503
CD11b	BV650	M1/70	BioLegend	101239
CD45	APC-R700	30-F11	BD Horizon	565478
F4/80	BV421	T45-2342	BD Horizon	565478
Ly6G	Percp-eflour 710	1AB-Ly6g	Invitrogen	46-9668-82
Intracellular:				
iNOS	PeCy7	CXNFT	Invitrogen	25-5920-80
ARG1	APC	A1exF5	Invitrogen	17-3697-82

**Table 4:** FACS antibodies for M1 and M2 macrophage staining.

Antibody against	Conjugated Fluorochrome	Clone Number	Commercial Source	Catalogue Number
Surface:				
CD11b	BV650	M1/70	BioLegend	101239
F4/80	Pe/Cy7	BM8	BioLegend	123114
CD80	FITC	16-10A1	BioLegend	104705
CD206	BV421	C068C2	BioLegend	141717

## Data Availability

All data presented within this study are available within the manuscript and the Supplementary Materials.

## Supplementary Materials

The following supporting information can be downloaded at: https://www.mdpi.com/article/10.3390/cells12081164/s1, Supplementary Materials: Table S1: Multireaction monitoring transitions for the six targets and the corresponding internal standards. Supplementary Figures: Figure S1: Gating strategy and regulation of M1 and M2 markers in macrophages due to stimulation and infection with *S*.tm; Figure S2: Measurement of cellular toxicity in infected BMDM pre-stimulated with different concentrations of IL-4 and IFNγ; Figure S3: Gene and protein expression of iNOS and ARG1 in uninfected and *S*.tm infected controls; Figure S4: Gating strategy of iNOS and ARG1 expressing macrophages and macrophages containing *S*.tm expressing RFP (STR); Figure S5: Pre- or post-stimulation of macrophages with IL-4 and IFNγ impacts on the amount of *S*.tm phagocytosed by F4/80+CD11b+ macrophages after 24 h of infection.
