# Supplementary material for: Timing of Interleukin-4 Stimulation of Macrophages Determines Their Anti-Microbial Activity during Infection with Salmonella enterica Serovar Typhimurium
Source: Cells. 2023 Apr 14;12(8):1164. doi: 10.3390/cells12081164 (PMC10137269; doi:10.3390/cells12081164)
Supplement: Supplementary file 1 [file cells-12-01164-s001.zip › cells-2148837-supplementary.pdf]

SUPPLEMENTAL INFORMATION

Supplementary Materials

Chemicals

Methanol, acetonitrile, and water (all HPLC grade) were obtained from Honeywell (Seelze, Germany). Triethylamine, phenyl isothiocyanate, acetic acid, putrescine, spermidine, spermine, L-arginine (as part of the amino acid standard A9906), as well as the internal standards arginine-$^{13}C_6$, methionine-$^{13}C_5$,^{15}N, and serine-$^{13}C3$,^{15}N (as part of the stable isotope labeled amino acid mix solution 96378) were purchased from Sigma-Aldrich (St. Louis, MO, USA). Putrescine-d8 and spermidin-d6 trihydrochloride were provided by Toronto Research Chemicals (Toronto, Ontario, Canada). To prepare solutions of putrescine, spermidine, spermine, putrescine-d8 and spermidin-d6, 1.0 mg of the standards were dissolved in methanol-water (1:1, v/v). For the preparation of calibration standards, the reference standard solutions were mixed and dissolved in methanol-water (1:1, v/v) to reach concentrations between 50 nM and 50 µM.

Derivatization procedure

10 µL of the cell lysates or calibration standards were pipetted into a 1.5 mL vial and mixed with 5 µL of the internal standard solution (2.5 µM of each internal standard in methanol-water (1:1, v/v)) as well as 65 µL methanol, 5 µL water, 10 µl triethylamine and 10 µL phenyl isothiocyanate. Derivatization was performed at room temperature for 30 min, after which, the excess reagent was removed by drying at 60 °C with a gentle stream of nitrogen. The dried samples were dissolved with 50 µL of methanol-water (3:1, v/v).

Liquid chromatography-tandem mass spectrometry

The LC-MS/MS system consisted of Acquity UPLC H-Class Plus Bio System (Waters, Milford, MA, USA) and a QTrap 6500+ mass spectrometer (Sciex, Framingham, MA, USA). Separations were accomplished on an Eclipse XBD-C18 column (3.5 µm, 3.0 × 100 mm, Agilent) using a ten-minute gradient of 2–98% acetonitrile in aqueous 0.5% acetic acid solution. The flow rate was set to 250 µL/min, and the column temperature was kept at 50 °C. The injection volume was 10 µL. Mass spectrometry detection was performed with electrospray ionization in positive ion mode. Multiple reaction monitoring was carried out using the precursor-to-product ion transitions summarized in Table S1. The peak area ratios of analyte-specific ions to the corresponding internal standard ions obtained from the calibration standards versus concentrations were used to fit 1/x−weighted linear, least squares regression models. The calibration models were used to calculate the analyte concentrations from the peak area ratios of analyte-specific ions to the corresponding internal standard ions obtained from the samples.

Table S1. Multireaction monitoring transitions for the six targets and the corresponding internal standards.

Target	Q1 (*m/z*)	Q3 (*m/z*)	Internal Standard
L-arginine	310	217	Arginine-$^{13}C_6$
Ornithine	403	310	Methionine-$^{13}C_5$,^{15}N
Putrescine	359	266	Putrescine-D8
Spermidine	551	193	Spermidine-D3

Spermine	743	193	Spermidine-D3
Arginine-$^{13}C_6$	316	223	
Methionine-$^{13}C_5$,^{15}N	291	109	
Serine-$^{13}C3$,^{15}N	245	63	
Putrescine-D8	367	274	
Spermidine-D3	557	199	

Supplementary Figures

(a)

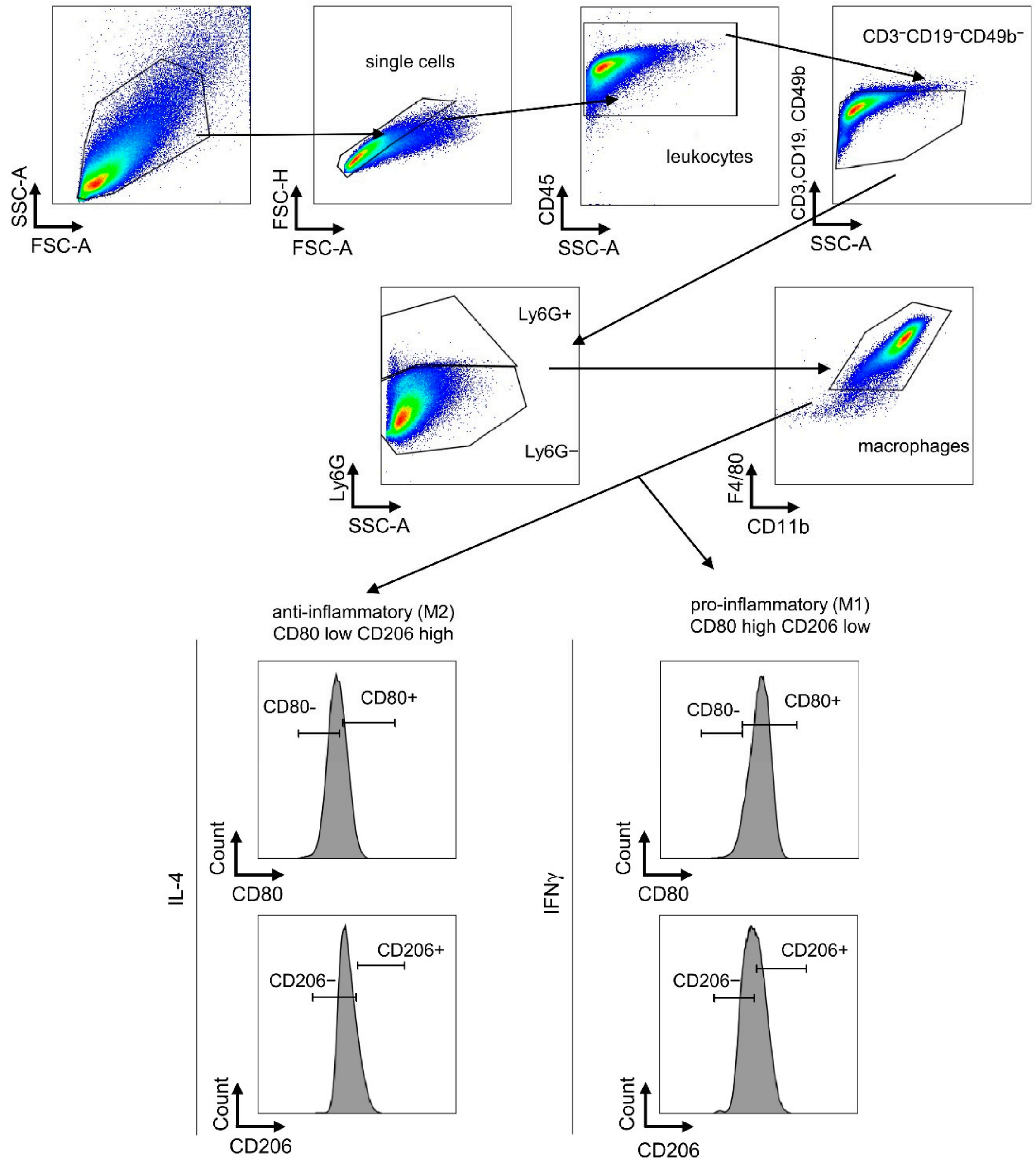

(b)

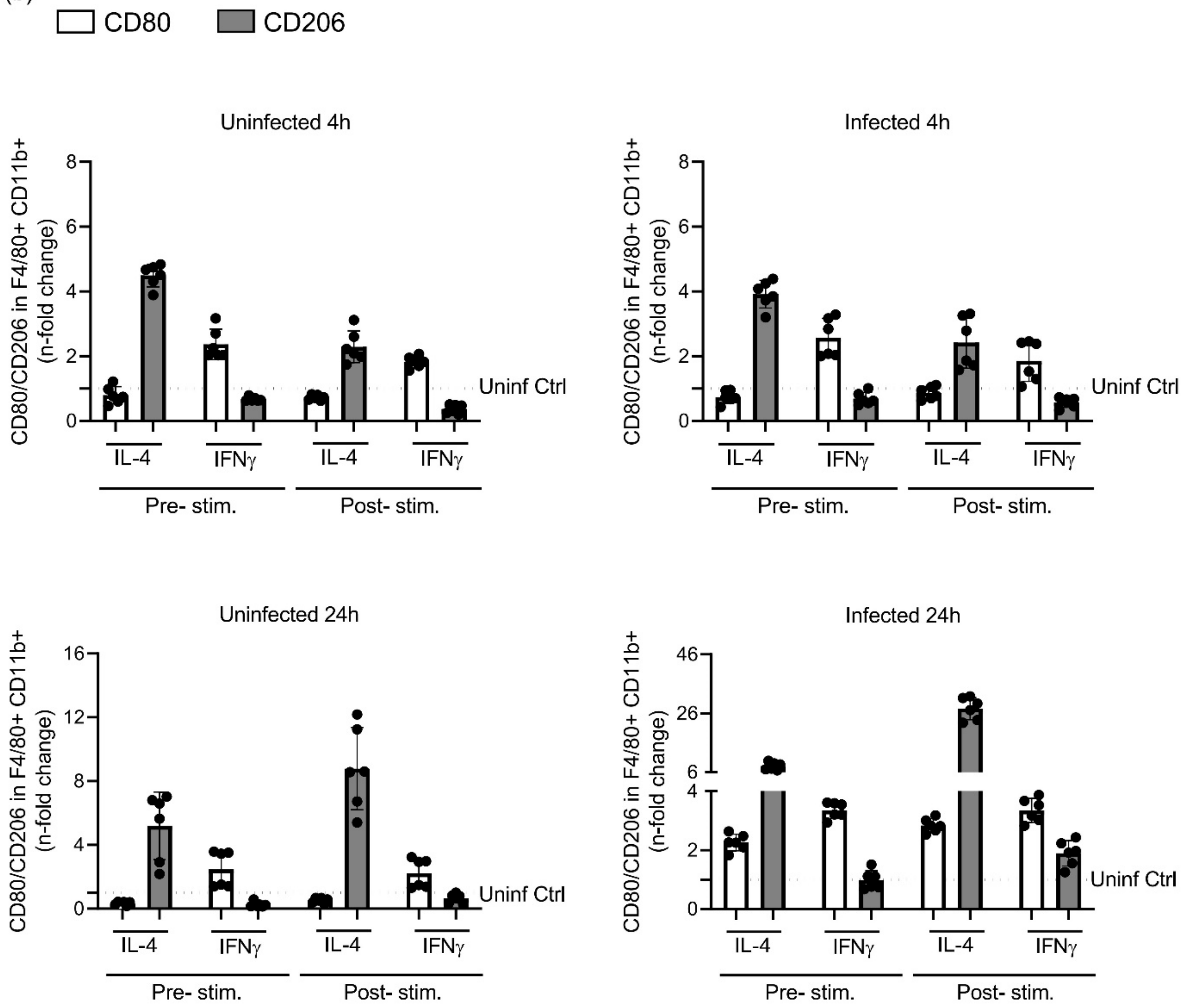

(c)

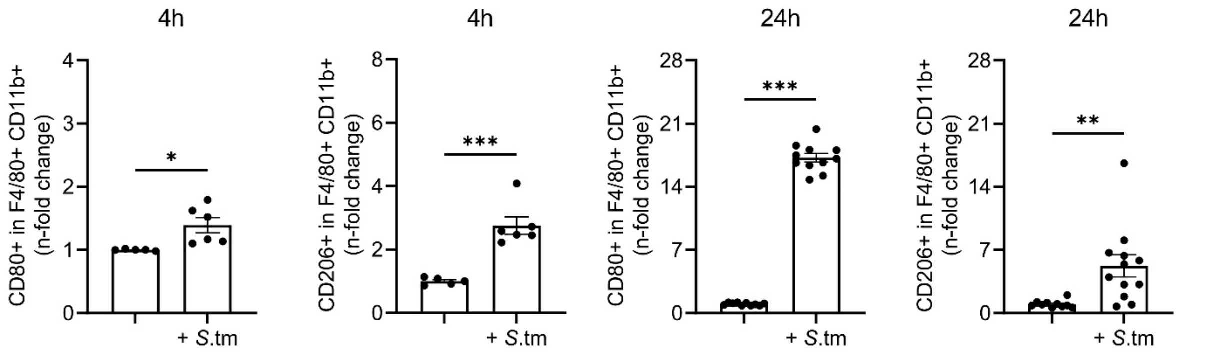

Figure S1: Gating strategy and regulation of M1 and M2 markers in macrophages due to stimulation and infection with *S*.tm.

Unpolarised BMDM and pre-stimulated polarised BMDM (2 ng/mL IL-4 or 20 ng/mL IFNγ) were infected with *S*.tm for 1h. Unpolarised cells were then stimulated (post-stimulation) with 10 ng/mL IL-4, 100 ng/mL IFNγ or left unstimulated as indicated. (a) Gating strategy to distinguish between pro-inflammatory (M1) and anti-inflammatory (M2) macrophages. (b) M1 and M2 macrophages in uninfected (left) and infected (right) pre- and post-stimulated conditions. M1 macrophages are described as F4/80^{+}CD11b^{+} CD80^{high}CD206^{low}, M2 macrophages are

described as F4/80^{+}CD11b^{+} CD80^{low}CD206^{high}. (c) Expression of CD80 and CD206 in F4/80^{+}CD11b^{+} BMDM uninfected and infected with S.tm after 4h and 24h.
Statistical significance was determined by Student's t-tests (b). * p-value < 0.05; ** p-value < 0.01; *** p-value < 0.001. Representative data (mean ± SEM) from three independent experiments with 2 technical replications are shown. Data were normalised to the unstimulated uninfected Ctrl.

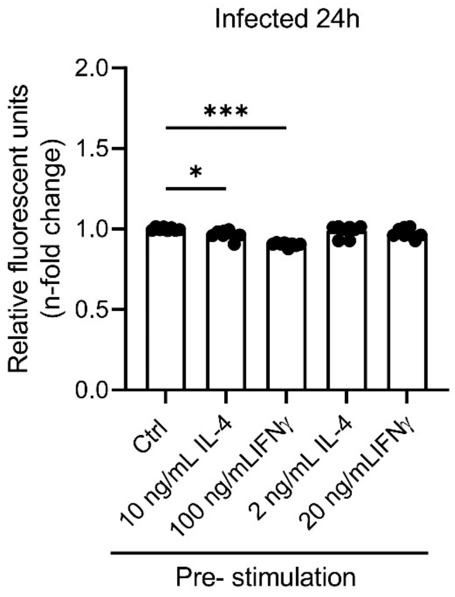

Figure S2: Measurement of cellular toxicity in infected BMDM pre-stimulated with different concentrations of IL-4 and IFNγ.
Cytotoxicity of IL-4 and IFNγ was tested by AlamarBlue assay in pre-stimulated BMDM (2 ng/mL IL-4, 20 ng/mL IFNγ or 10 ng/mL IL-4, 100 ng/mL IFNγ). BMDM were infected with *S*.tm for 1h. Supernatant was taken after 24h. Data were normalised to the unstimulated infected Ctrl. Statistical significance was determined by one-way ANOVA with Tukey post hoc test: * p-value < 0.05; ** p-value < 0.01; *** p-value < 0.001. Representative data (mean ± SEM) from three independent experiments with 2 technical replications are shown.

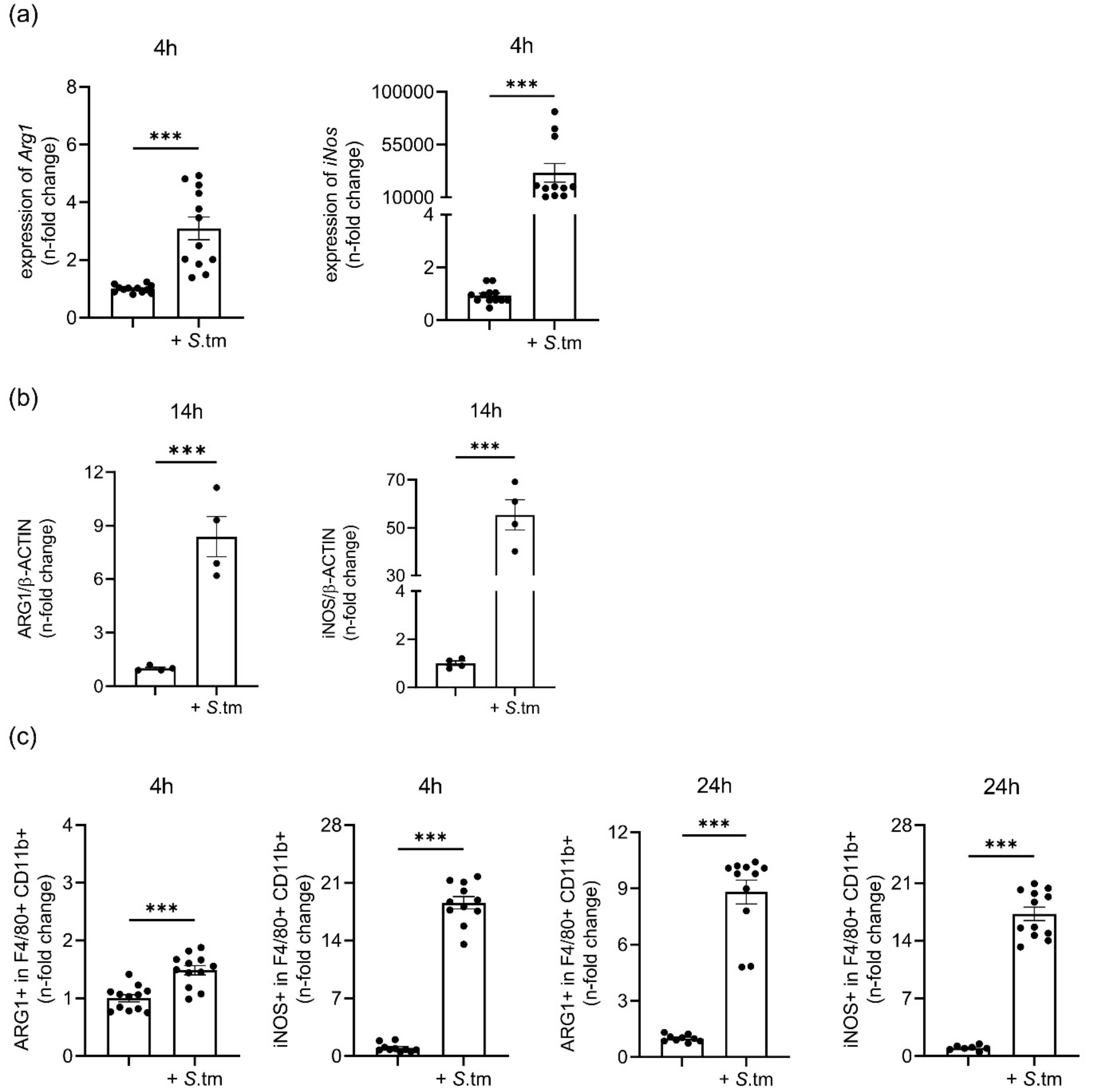

Figure S3: **Gene and protein expression of iNOS and ARG1 in uninfected and *S*.tm infected controls.**

BMDM were either infected with *S*.tm for 1h or left uninfected and analysed after 4h and 24h. (a) *Arg1* (left) and *iNos* (right) transcript levels due to infection were determined by quantitative real-time PCR and normalized to Hypoxanthine phosphoribosyltransferase (*Hprt*) mRNA levels using the ΔΔCT method. (b) Densidometrical quantification of immunoblotting analysis of ARG1 (left) and iNOS (right) relative to β-ACTIN. (c) Percentage of ARG1 and iNOS positive cells in F4/80$^+$CD11b$^+$ cells at different time points of infection.

Statistical significance was determined by Student's t-tests. *** p-value < 0.001. Representative data (mean ± SEM) from three independent experiments are shown.

Data were normalised to the unstimulated uninfected Ctrl.

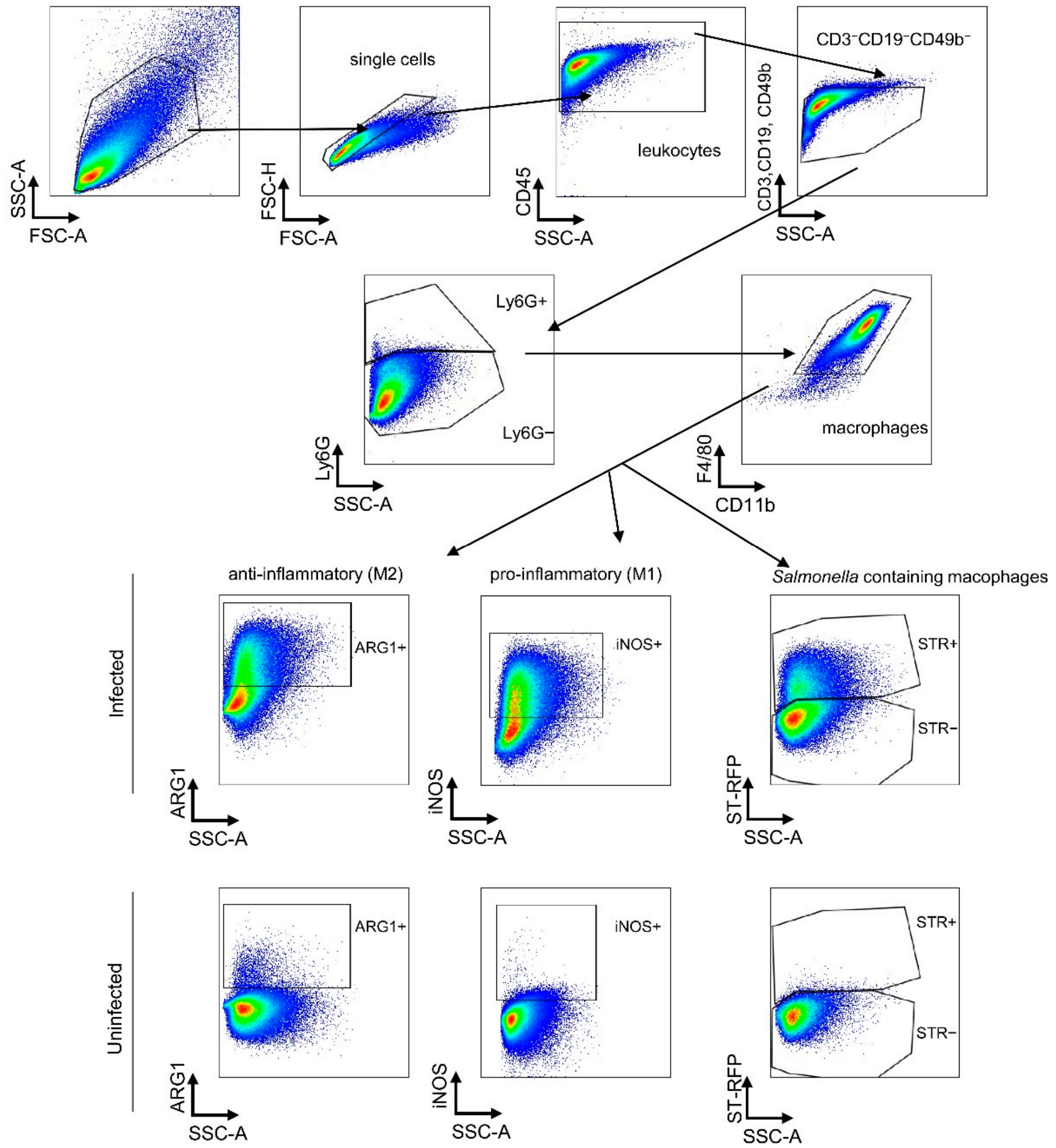

Figure S4: Gating strategy of iNOS and ARG1 expressing macrophages and macrophages containing *S*.tm expressing RFP (STR).

BMDM were infected with *S*.tm expressing RFP for 1h and stained with surface markers and intracellular markers. Doublets were excluded and $CD45^+$ cells were analysed. $CD3^+$, $CD19^+$ and $CD49^+$ cells as markers for T cells, B cells, and NK cells were excluded. $Ly6G^-$ cells were analysed for $CD11b^+F4/80^+$ macrophage populations. These cells were further analysed for iNOS and ARG1 positive macrophage populations and for macrophages containing RFP expressing *Salmonella*.

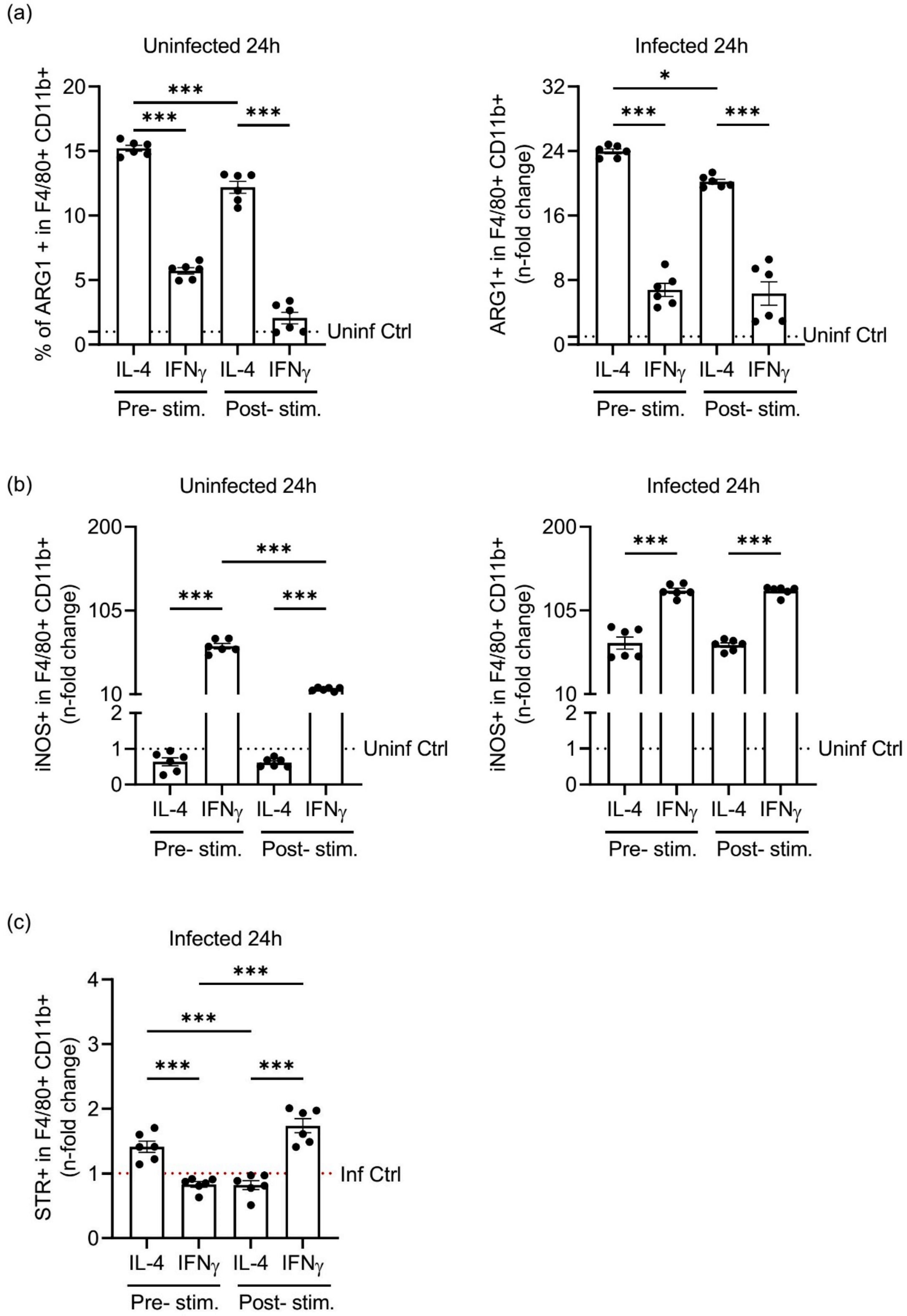

Figure S5: Pre- or post-stimulation of macrophages with IL-4 and IFNγ impacts on the amount of S.tm phagocytosed by F4/80$^+$CD11b$^+$ macrophages after 24h of infection. Unpolarised BMDM and pre-stimulated polarised BMDM (2 ng/mL IL-4 or 20 ng/mL IFNγ) were infected with *S*.tm expressing RFP for 1h. Unpolarised cells were then stimulated (post-stimulation) with 10 ng/mL IL-4, 100 ng/mL IFNγ or left unstimulated for 24h. Percentages of ARG1, iNOS and STR were analysed by flow cytometry. (a) Percentage of ARG1 positive cells in F4/80$^+$CD11b$^+$ cells in uninfected (left) or infected (right) pre-stimulation compared to post-

stimulation conditions. (b) Percentage of iNOS positive cells in F4/80$^+$ CD11b$^+$ cells in uninfected (left) or infected (right) pre-stimulation compared to post-stimulation conditions. (c) Percent of STR positive cells in F4/80$^+$ CD11b$^+$ cells in infected pre-stimulation compared to post-stimulation conditions.

Statistical significance was determined by one-way ANOVA with Tukey post hoc test. * p-value < 0.05; *** p-value < 0.001. Representative data (mean ± SEM) from three independent experiments with 2 technical replications are shown.

Data were normalised to the unstimulated uninfected Ctrl or, were indicated, to the unstimulated infected Ctrl.
